# Metal-responsive promoter DNA compaction by the ferric uptake regulator

**DOI:** 10.1038/ncomms12593

**Published:** 2016-08-25

**Authors:** Davide Roncarati, Simone Pelliciari, Nicola Doniselli, Stefano Maggi, Andrea Vannini, Luca Valzania, Luca Mazzei, Barbara Zambelli, Claudio Rivetti, Alberto Danielli

**Affiliations:** 1 Department of Pharmacy and Biotechnology (FaBiT), University of Bologna, 40126 Bologna, Italy; 2 Department of Life Sciences, University of Parma, 43124 Parma, Italy

## Abstract

Short-range DNA looping has been proposed to affect promoter activity in many bacterial species and operator configurations, but only few examples have been experimentally investigated in molecular detail. Here we present evidence for a metal-responsive DNA condensation mechanism controlled by the *Helicobacter pylori* ferric uptake regulator (Fur), an orthologue of the widespread Fur family of prokaryotic metal-dependent regulators. *H. pylori* Fur represses the transcription of the essential *arsRS* acid acclimation operon through iron-responsive oligomerization and DNA compaction, encasing the *arsR* transcriptional start site in a repressive macromolecular complex. A second metal-dependent regulator NikR functions as nickel-dependent anti-repressor at this promoter, antagonizing the binding of Fur to the operator elements responsible for the DNA condensation. The results allow unifying *H. pylori* metal ion homeostasis and acid acclimation in a mechanistically coherent model, and demonstrate, for the first time, the existence of a selective metal-responsive DNA compaction mechanism controlling bacterial transcriptional regulation.

## Abstract (web summary)

The Fur protein regulates transcription of bacterial genes in response to metal ions. Here, the authors show that the Fur protein from *Helicobacter pylori* represses transcription by iron-responsive oligomerization and DNA compaction, encasing the transcriptional start site in a macromolecular complex.

## Graphical abstract

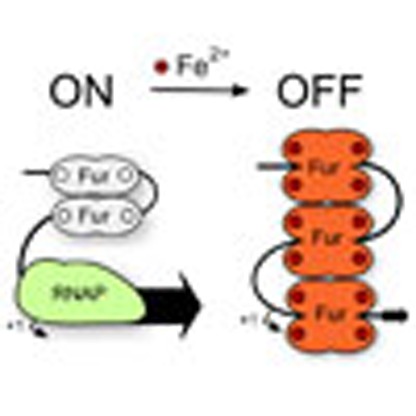

DNA condensation and looping are fundamental mechanisms for genome biology and gene regulation in prokaryotes and eukaryotes. In bacteria, long-range DNA compaction and gene expression impact on the topological organization of the nucleoid^1^, while local DNA bending by transcription factors and nucleoid-associated proteins (NAPs) has provided early paradigmatic evidence for the importance of DNA looping in the regulation of promoter elements^2^,^3^.

Accordingly, short-range DNA looping has been proposed to account for particular operator configurations and promoter responses in countless reports and many bacterial species. Nevertheless, only few examples have been experimentally investigated in molecular detail, principally, but not exclusively, involving phage repressors^4^, and the sugar, nitrogen or nucleotide catabolism regulation in *Escherichia coli*^5^,^6^,^7^,^8^. The capability to constrain and to locally distort the DNA is also a key feature of NAPs and global regulators involved in transcriptional control^9^. Remarkably, the involvement of transcription factor-mediated DNA looping in the transduction of metal-dependent transcriptional responses has not been reported to date, although several metal ions and metal-sensing regulators (such as Fe^2+^-Fur and Ni^2+^-NikR) have fundamental roles in bacterial viability, virulence and host–pathogen interactions^10^. A unique exception pertains to the recent prediction of a putative Fur-dependent DNA looping at the *fepB-entCEBAH* promoter, inferred from theoretical binding site annotations of *E. coli* promoters^6^. Fur proteins belong to an ubiquitously conserved superfamily of prokaryotic regulators involved in the homeostasis of different metal ions and oxidative stress responses^11^. Because of their ability to oligomerize in a metal-dependent fashion^12^,^13^ and to bind promoters at multiple sites^14^,^15^,^16^, they represent ideal candidates for the investigation of metal-dependent DNAe. In addition, topological modifications of the DNA induced by Fur binding, such as bending and wrapping, have been reported *in vitro* using various footprinting and microscopy techniques, including atomic force microscopy (AFM)^17^,^18^.

Herein, we use the thoroughly characterized *H. pylori* Fur regulator as a model to explore the metal-dependent short-scale DNA compaction mechanisms involved in transcriptional regulation. To this aim, we investigate the Fur-regulated *arsR* promoter by a combination of DNase I footprinting, AFM and promoter functional analysis. The rationale behind the choice of this promoter derives from the ChIP-chip and functional evidence that Fur regulates and targets the *arsRS* operon *in vivo*^19^,^20^. This operon encodes a two-component system that controls transcription of many *H. pylori* pH-dependent genes through the activity of the auto-regulated ArsR response regulator^21^. ArsR is crucial for the positive regulation of the nickel-dependent urease metallo-enzyme, which is important for acid acclimation^22^. This transcriptional regulator also appears to positively regulate other essential pH-independent functions of *H. pylori* because, unlike the *arsS* histidine kinase gene, *arsR* deletion mutants have not been attainable to date. Interestingly, the acid tolerance of *H. pylori* was shown to be impaired in both *fur* and *nikR* (nickel-dependent regulator) deletion mutants^23^,^24^, fostering the hypothesis of a shallow regulatory circuit linking metal-ion homeostasis with acid acclimation^25^.

In this work, we characterize the role of these metal-dependent regulators in the transcriptional control of the essential *arsRS* operon. We demonstrate the direct wiring of Fur and NikR with the ArsR regulon, and highlight the contribution of DNA compaction to this process. In particular, we show that the *arsR* promoter sports a complex operator architecture with multiple Fur and NikR operators, bound with different affinities according to the metallation state of the regulators. Evidence is presented that this promoter architecture allows for iron- and Fur-dependent repression (FeOFF) through the compaction of the promoter in a nucleo–protein complex. In the presence of nickel ions, binding of NikR antagonizes Fur binding and DNA compaction thus relieving Fur repression (NikRON). The fundamental implications of this metal-responsive promoter compaction mechanism are discussed, together with its pivotal role in the *H. pylori* transcriptional regulatory circuit.

## Results

### ArsR transcription is controlled by Fur and NikR metallo-regulators

**Figure 1:**
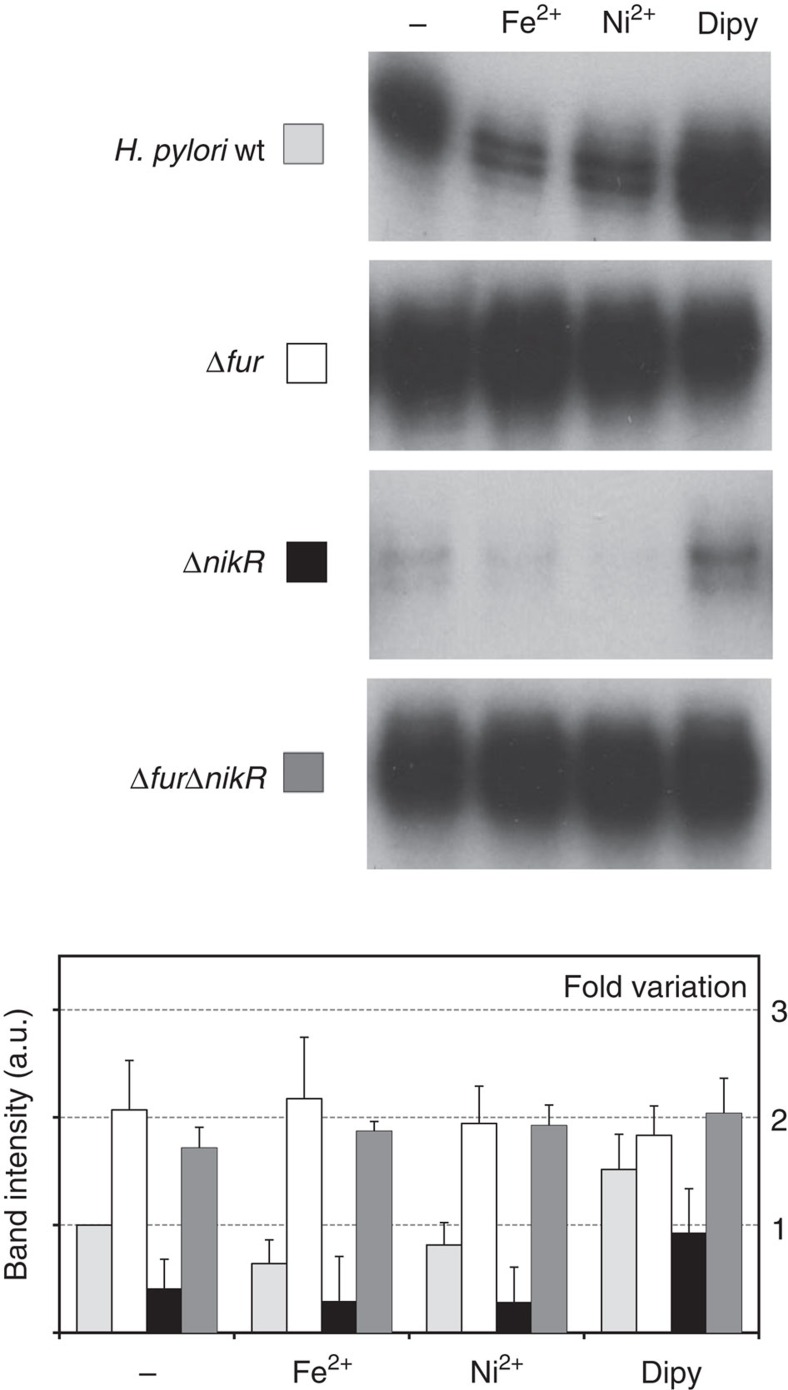
Transcriptional analysis of the *arsR* gene in response to metal ion treatment. Primer extensions performed on total RNA extracted from wild-type, Δ*fur,* Δ*nikR* and Δ*fur*Δ*nikR* double mutants *H. pylori* strains grown to exponential phase and treated for 15 min with 1 mM (NH_4_)_2_Fe(SO_4_)_2_ (Fe^2+^), 1 mM NiSO_4_ (Ni^2+^) or 100 μM 2-2 dipyridyl (Dipy); untreated control RNA (−). Full gel is shown in Supplementary Fig. 6. Fold variation of the band intensities is reported in the graph. Error bars represent the standard deviation recorded in four independent experiments.

To investigate how metal homeostasis regulation could transcriptionally feed into the ArsR regulon, we performed primer extension analyses of the native P*arsR* promoter using total RNA extracted from exponential phase *H. pylori* cultures and we compared the responses elicited by treatment with different metal ions (Fe^2+^ and Ni^2+^) or iron chelator (Dipy), under different genetic backgrounds (Fig. 1).

In a wild-type background, P*arsR* transcription was repressed by either iron or nickel, while iron chelation caused de-repression of the promoter, pointing to a prominent role of Fur in *arsR* regulation. Accordingly, P*arsR* was constitutively de-repressed in a *fur* knockout strain, suggesting a prototypical holo-Fur repression in which the iron ion acts as co-repressor (FeOFF). While the slight repression observed after nickel treatment could indicate a repressive role of NikR on P*arsR*, we observed a generalized decrease of *arsR* transcript levels in the Δ*nikR* strain, in which the responses to metal-ions and chelators were maintained. At a first glance, this could be interpreted as a NikR-dependent transcriptional activation of P*arsR*; however, while the responses in wild-type and *fur*-deletion mutants proved highly reproducible, the Δ*nikR*-dependent deregulation of P*arsR* was more variable. In a Δ*furΔnikR* double mutant background, the *ars* promoter remained constitutively de-repressed, excluding the requirement of NikR for full promoter activation, and suggesting that the observed nickel-induced repression is directly or indirectly mediated by Fur, which would be responsible for the transduction of the Ni^2+^ signal. The documented ability of Ni^2+^ to substitute Fe^2+^ as Fur cofactor in footprinting experiments^14^ supports this interpretation.

These results demonstrate that the essential ArsR acid response regulator is under the transcriptional control of the metal ion circuit regulated by Fur and NikR, with the former acting as metal-responsive master repressor (FeOFF) and the latter as positive modulator of *arsR* transcription.

### Complex architecture of the *arsR* promoter

**Figure 2:**
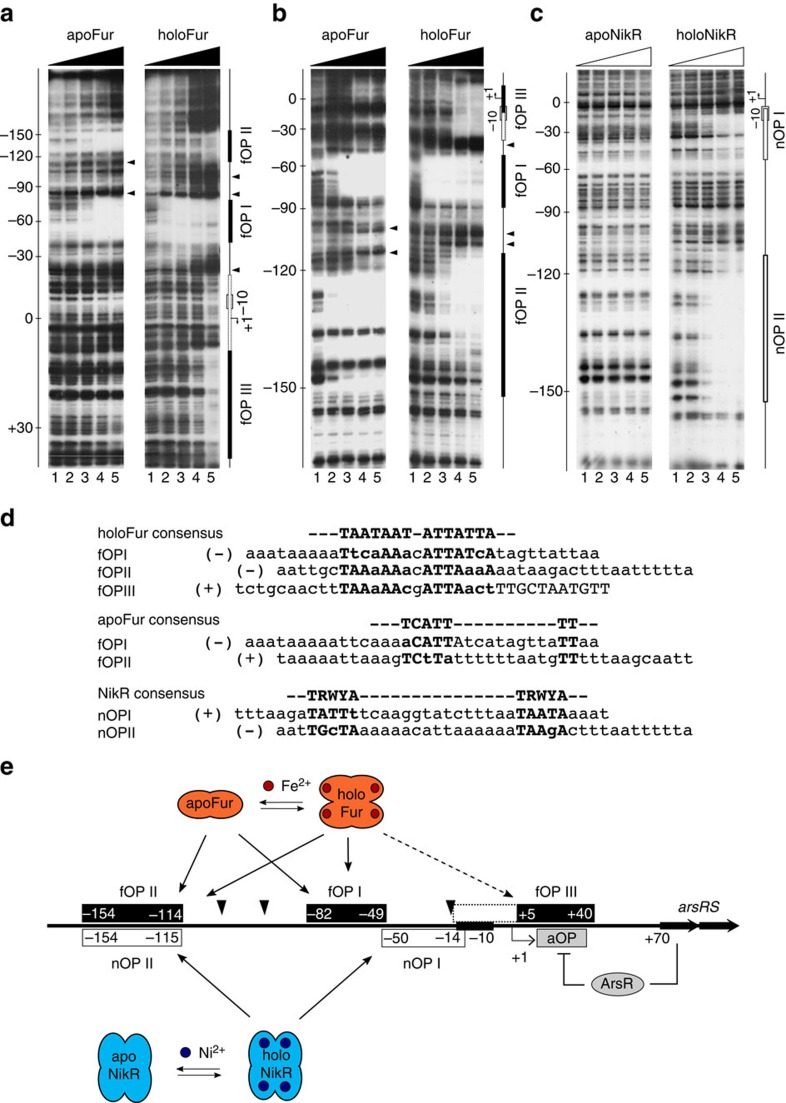
DNA footprinting of apo/holo-Fur and apo/holo-NikR at P*arsR*. (**a**) apo-Fur (left gel) and holo-Fur (right gel) on the coding strand; (**b**) apo-Fur (left gel) and holo-Fur (right gel) on noncoding strand; (**c**) apo-NikR (left gel) and holo-NikR (right gel) on noncoding strand. Lanes 1–5: 0, 35, 70, 140 and 280 nM monomeric Fur or NikR, respectively. The scale bar on the left of each gel shows the distance from the TSS. A schematic representation of the promoter is presented on the right side of each gel with Fur and NikR footprints outlined as black and white boxes, respectively. Black arrowheads indicate persistent or hypersensitive bands. (**d**) Comparison of the Fur and NikR operator sequences on P*arsR* with the previously defined consensus motifs of the regulators. (**e**) Inferred schematic representation of the operator layout in the P*arsR* promoter. Fur operators named fOPI, fOPII and fOPIII are depicted as black boxes, while NikR operators named nOPI and nOPII are depicted as white boxes. The position of the ArsR operator aOP is mapped as reported in ref. 26. Positions are indicated with respect to the TSS.

To elucidate the molecular mechanisms responsible for the regulation of *arsR*, the protein–DNA interactions of recombinant Fur and NikR at the P*arsR* promoter were investigated by DNase I footprinting (Fig. 2).

Fur exhibited a complex pattern of iron-sensitive (apo-) and iron-dependent (holo-) operators: when iron was chelated from the reaction, the resulting apo-Fur protein protected two distinct elements (Fig. 2a; left gel): (i) a distal high affinity apo-operator (fOPII; full protection at 30 nM apo-Fur) located from −114 to −154 bp upstream of the transcriptional start site (TSS) and (ii) a central operator (fOPI) extending from −49 to −82 bp upstream of the TSS (protection at 75 nM apo-Fur). When Fur was pre-incubated with iron the affinities for these operators changed (Fig. 2a; right gel). In particular, while the affinity for the distal apo-operator decreased, the affinity for the central operator increased 5-fold (protection at 12 nM holo-Fur). In addition, a third low-affinity element (fOPIII) appeared beneath and immediately downstream of the TSS (protection at 125 nM holo-Fur). To better define the interactions of Fur within the promoter region, apo- and holo-Fur DNA footprinting was performed with a P*arsR* probe labelled on the noncoding (antiparallel) strand (Fig. 2b). The observed protection patterns matched closely those obtained on the coding strand, mapping the principal holo-Fur protection from +5 to +40 bp downstream of the *arsR* TSS, with a weaker but reproducible upstream extension of the footprint into the core promoter. This low affinity holo-Fur element overlaps the core promoter and the ArsR operator^26^ required for auto-repression by ArsR.

Notably, distinct hypersensitive and/or persistent bands appeared upon Fur binding under different metallation states (see arrowheads in Fig. 2). When iron was chelated two persistent bands, encompassed between the central (fOPI) and the distal apo-Fur operators (fOPII), appeared. Upon binding of holo-Fur, these persistent bands repositioned more closely, while a strong hypersensitive band became visible between the central (fOPI) operator and the downstream low affinity holo-Fur operator (fOPIII), indicating bending of the DNA in this region upon Fur binding. In addition, another two bands distal to fOPII appeared stronger with increasing amounts of Fur, which may be imputable to an overrepresentation of longer DNA fragments due to inhibition of DNAse I by higher conentrations of Fur. These results suggest the existence of different DNA topologies determined by the binding of apo- or holo-Fur to P*arsR*.

The pattern of NikR interactions with the P*arsR* probe proved more straightforward. In the absence of Ni^2+^ ions (Fig. 2c; left gel), apo-NikR was unable to bind DNA, in accordance with previous reports^27^. When NikR was preincubated with nickel (Fig. 2c; right gel), two regions of DNase I protection appeared at approximately similar NikR concentrations (protection at 40–60 nM holo-NikR). The distal element (nOPII) (from −115 to −154) perfectly overlapped the distal apo-Fur operator, while the proximal NikR element (nOPI; from −14 to −50) mapped adjacent and downstream of the central Fur operator. In contrast to Fur, binding of NikR to the P*arsR* probe did not elicit DNase I hypersensitive bands.

Overall, the regions protected by holo-Fur, apo-Fur and NikR match well to the previously identified consensus sequences^12^,^28^ (Fig. 2d) and reveal a complex architecture of the *arsR* promoter region, which can be schematized as shown in Fig. 2e. It will be noted that Fur and NikR operators are counterintuitively positioned on the *ars* promoter with regard to their regulatory function. In particular, the Fur-iron repression of P*arsR* observed in primer extension experiments, would suggest at least one high affinity holo-Fur operator overlapping the core promoter. Actually, the high affinity Fur operators (fOPI and fOPII) map further upstream, in positions usually occupied by class I activators, whereas only a low affinity element (fOPIII) is found in a position compatible with promoter occlusion and transcriptional repression. A similar discrepancy applies for NikR, as the nOPI operator maps immediately upstream of the −10 box, in a position frequently occupied by transcriptional repressors, albeit NikR acts as a positive regulator of P*arsR*.

### Distinct macromolecular assemblies upon Fur and NikR binding to P*arsR*

The positions of persistent and hypersensitive bands observed upon binding of Fur to DNA, indicate that the metal-dependent transcriptional responses of *arsR* could derive from different macromolecular conformations of the *arsR* promoter region, shaped by Fur or NikR binding. To ascertain this hypothesis, AFM experiments were carried out to capture the structural details conferred by the binding of these regulators to the P*arsR* promoter region.

First, we analysed the oligomerization state of apo- and holo-Fur in the absence of DNA. To this end, we constructed a volume calibration curve using proteins of known molecular weight (Supplementary Fig. 1). Deposition of both apo- and holo-Fur onto freshly cleaved mica, followed by AFM imaging, resulted in a similar distribution of monomers and dimers (Supplementary Fig. 2A,B). This result was unexpected because previous reports indicated that apo-Fur is mainly a dimer while holo-Fur is prevalently represented by tetramers or higher order oligomers^12^. Because the high surface charge of mica can readily disrupt the protein quaternary structure, we used glutaraldehyde crosslinking to fix the Fur oligomeric states. Under these conditions, apo-Fur mostly comprised monomers and dimers even though the dimeric state is more populated than the monomeric one and a discrete number of particles have an inferred mass of tetramers (Supplementary Fig. 2C). Conversely, the oligomers formed by holo-Fur comprise dimers, tetramers, octamers and even higher oligomeric states (Supplementary Fig. 2D). This analysis confirms previous observations of the iron effect on the stoichiometry of Fur, and validate the application of AFM for the characterization of these complex macromolecular assemblies.

**Figure 3:**
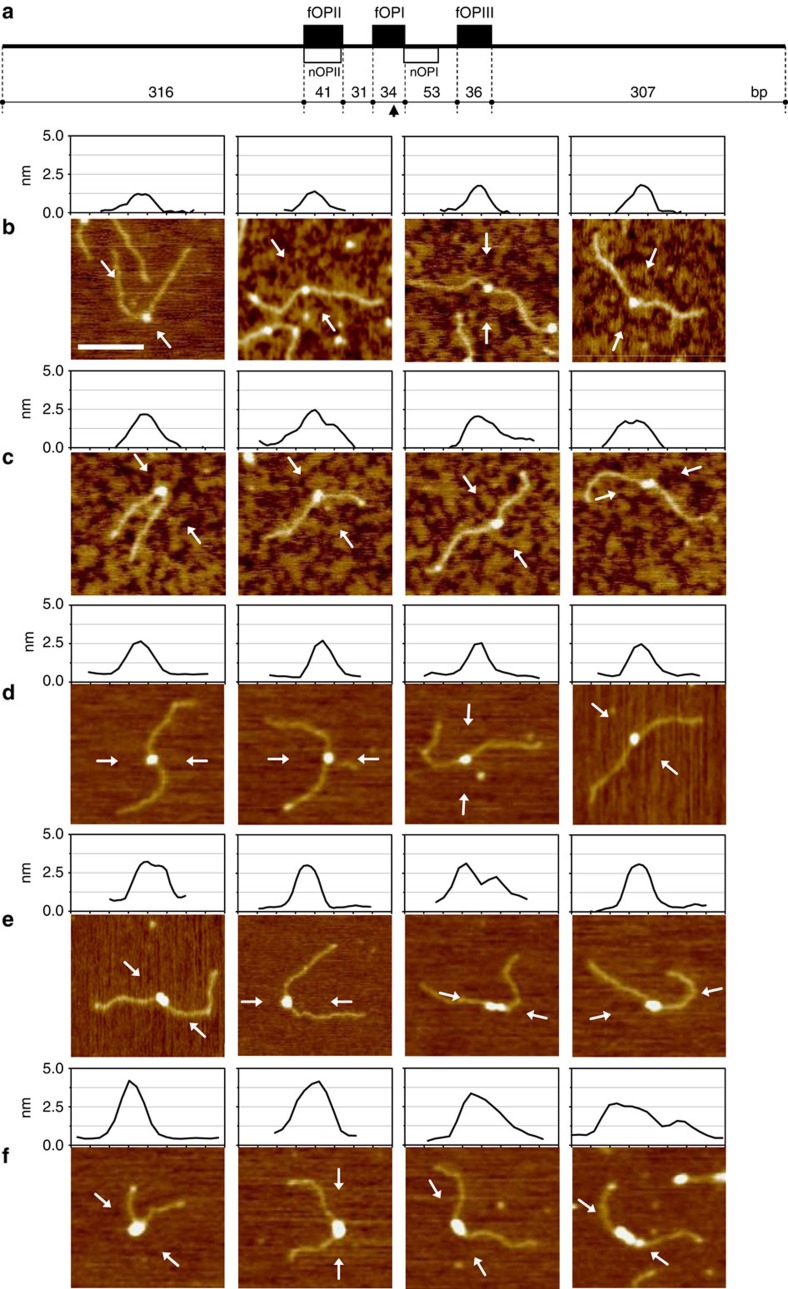
AFM images of Fur–DNA complexes. (**a**) Scaled representation of the 818 bp DNA template used in the AFM experiments. Fur operator sites (black boxes) and NikR operator sites (white boxes) are indicated. Distances are in base pairs and the arrow indicates the midpoint of the template. (**b**) One apo-Fur dimer bound to the central fOPI site. (**c**) Two apo-Fur dimers bound to fOPI and fOPII sites. (**d**) One holo-Fur tetramer bound to the central fOPI site. (**e**) Two holo-Fur tetramers bound to fOPI and fOPII sites. (**f**) Three or more tetramers bound to fOPI, fOPII and fOPIII sites with consequent large DNA compaction. The image profile along the direction indicated by white arrows is shown on top of each panel. Scale bar, 100 nm. The profile plots have a width of 80 nm.

Next we analysed the DNA-binding properties of apo- and holo-Fur to a 818 bp fragment harbouring the P*arsR* promoter region (Fig. 3a). From the binding of apo-Fur to P*arsR*, we observed two different categories of complexes: one characterized by a small globular feature with an average volume of 123±33 nm^3^ (Fig. 3b) and the other characterized by a larger globular feature with an average volume of 270±56 nm^3^ (Fig. 3c). By means of the calibration curve, we could infer that the former complexes correspond to a particle with a MW of 56±21 kDa, which fits well the molecular mass of a Fur dimer (35 kDa) bound to 34 bp of DNA (22 kDa) (Table 1). Analysis of the DNA contour length of these complexes further indicates that Fur dimers are bound closely to the centre of the DNA template, in a position corresponding to the fOPI site. Furthermore, the DNA contour length is not altered by the binding of Fur (Table 1), thus suggesting the absence of a large protein-induced DNA deformation^29^. The second category of apo-Fur–DNA complexes is characterized by globular features with a larger volume and a higher image profile, bound to the DNA template slightly off centre. The inferred mass of these complexes is 113±30 kDa (Table 1), in line with a nucleoprotein complex of two apo-Fur dimers (70 kDa) bound to about 100 bp of DNA, comprising fOPI and either fOPII or fOPIII. However, on the basis of the footprinting results, binding of apo-Fur to fOPIII can be disregarded. In some images, this particular arrangement is supported by the observation of a double peak in the image profile (Fig. 3c; panels 2 and 4). Interestingly, the DNA contour length analysis of these complexes reveals a decrease of 17.4±2.5 nm suggesting that most, if not all, the bound DNA is compacted within the nucleo–protein complex.

AFM analysis of the holo-Fur interaction with the P*arsR* operator sites revealed remarkably different type of complexes, which were subdivided into three categories with features summarized in Table 1. The first category (Fig. 3d) comprises complexes with a volume of 220±39 nm^3^ and an inferred molecular mass of 93±24 kDa, which well fits the molecular mass of one holo-Fur tetramer (70 kDa) bound to 34 bp of DNA (22 kDa). As before, the position of the complex indicates binding to the fOPI site. Notice that, in contrast to the two apo-Fur dimers shown in Fig. 3c, the image profiles reveal complexes formed by a compact globular feature with a single (slightly higher) peak. DNA contour length measurements indicate that binding of the tetramer to fOPI results in a DNA compaction of 7.4±3 nm (23±9 bp). This small DNA compaction can arise from the different path that the DNA can take upon deposition (see legend to Supplementary Fig. 2).

The second type of complexes formed by holo-Fur at the P*arsR* promoter is characterized by globular features with a volume of 568±136 nm^3^ that corresponds to a molecular mass of 228±61 kDa. These complexes are most likely formed by two holo-Fur tetramers (140 kDa) bound to fOPI and fOPII and thus including more than 100 bp of DNA, which in terms of mass can account for additional 70–80 kDa. The DNA contour length analysis reveals a decrease of 14.9±1.8 nm suggesting, as in the case of the two apo-Fur dimers, that contact between the two tetramers determines a significant DNA compaction, an observation supported by the dumb-bell shape of the complexes captured in a few cases (Fig. 3e).

Finally, the third category of complexes is represented by very large globular features bound near the centre of the DNA template, which appears visibly shortened (Fig. 3f). These complexes have an average volume of 1,089±244 nm^3^ and an inferred mass of 430±103 kDa. The DNA contour length compaction is of 65.4±10.8 nm, corresponding to 204±34 bp of DNA in agreement with the 195 bp DNA region spanned by the three operators fOPI, fOPII and fOPIII. Therefore, we propose that these complexes are formed by three holo-Fur tetramers each bound to one of the three operator sites. Given the tendency of holo-Fur to oligomerize, protein–protein interaction between the tetramers condenses the P*arsR* promoter region into a large nucleo–protein complex. In few cases, the particular deposition of the complex makes it possible to distinguish three individual tetramers bound to this promoter region (Fig. 3f; last panel).

The AFM experiments described above have been performed using complexes crosslinked with glutaraldehyde to prevent oligomers dissociation upon deposition onto mica. To verify that this treatment does not lead to artifactual results, we imaged the 818 bp bare DNA fragment incubated with 10 mM glutaraldehyde either for 2 or 10 min (Supplementary Fig. 3A,B). Under these conditions, we did not observe significant changes of the DNA shape, indicating that crosslinking does not affect the overall structure of the DNA. Furthermore, to assess that the higher order Fur oligomerization observed in the presence of iron is due to the peculiar architecture of the P*arsR* promoter, we constructed a mutated variant by inserting a 315 bp spacer between the fOPI and fOPII sites to drastically change their distance. This mutant was designed also with the aim to visualize a possible DNA loop that would be formed by the interaction of Fur bound to the distant operator sites. Under these conditions, we observed complexes prevalently bound to fOPI, with an image profile height and volume consistent with a holo-Fur tetramer. Neither DNA looping nor higher order oligomers were observed (Supplementary Fig. 3C,D). These results demonstrate that the oligomeric states of holo-Fur bound to the P*arsR* promoter and the consequent DNA compaction are determined by the unique architecture of this promoter and not by artifacts introduced with the crosslinking procedure.

**Figure 4:**
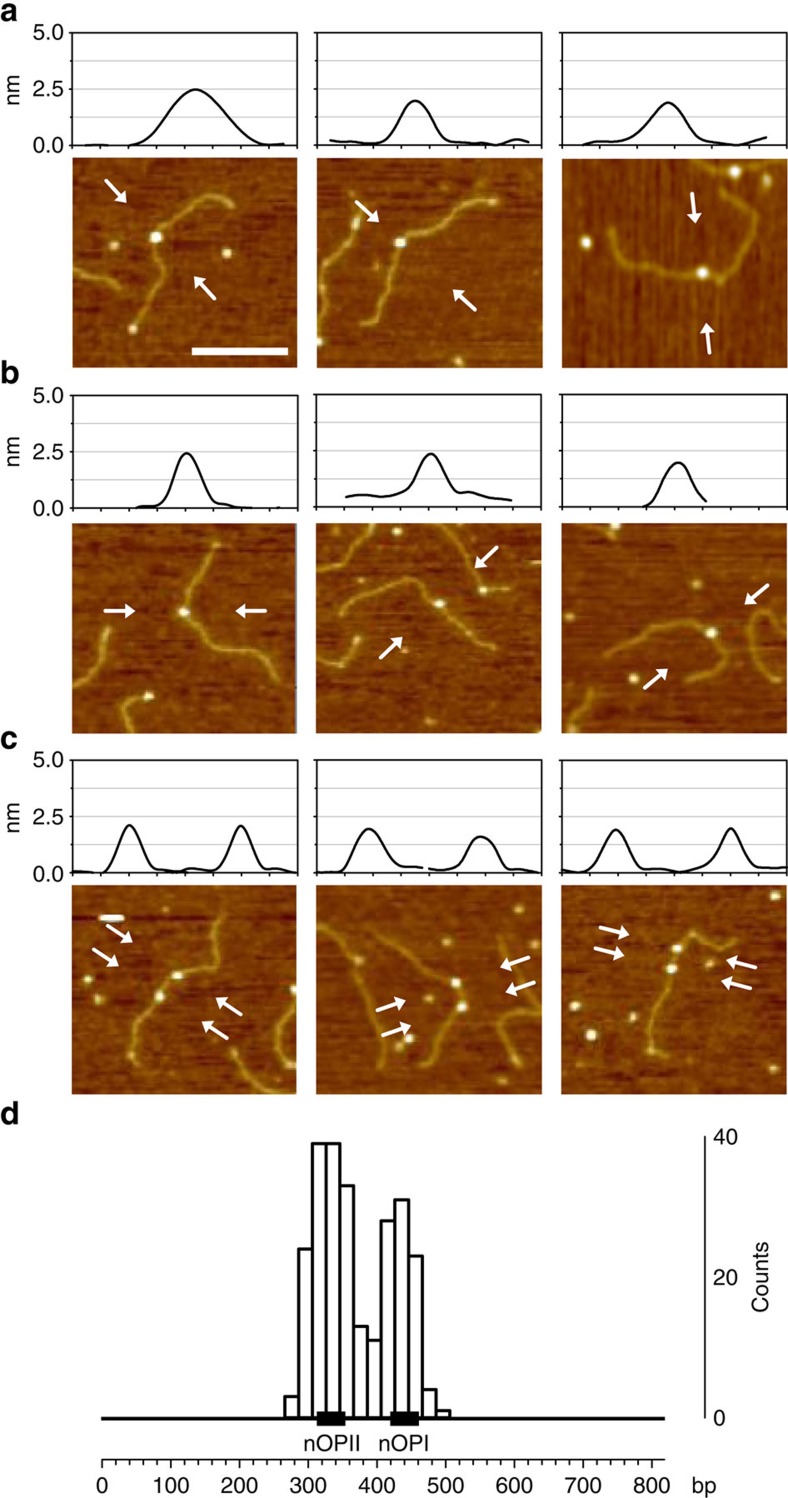
AFM images of holo-NikR–DNA complexes. The image profile along the direction indicated by white arrows is shown on top of each panel. (**a**) One holo-NikR tetramer bound to the central nOPI site. (**b**) One holo-NikR tetramer bound to the slightly off-centre nOPII site. (**c**) Two holo-NikR tetramers bound to both nOPI and nOPII sites. (**d**) Bar chart representing the position of NikR bound along the DNA template (black line) with the nOPI and nOPII sites represented, in scale, as black boxes. Graph scale in base pairs. Scale bar, 100 nm. Width of the profile plot, 80 nm.

The AFM analysis was then applied to investigate binding of NikR to the *Pars*R promoter. Because apo-NikR does not bind DNA (Fig. 2c), the analysis was carried out only at saturating Ni^2+^ concentrations (1 mM). Under these conditions, holo-NikR forms stable tetramers that do not oligomerize in particles of high molecular mass^27^. For complexes assembly, we used the 818 bp-long DNA fragment described above as it harbours also two NikR operator sites (nOPI and nOPII) separated by 65 bp (103 bp, centre-to-centre; Fig. 2d). AFM images of NikR–DNA complexes showed that holo-NikR binds frequently near the centre of the DNA template, either on nOPI or nOPII (Fig. 4a,b, respectively), and in a few cases we could observe two NikR bound to the same DNA molecule (Fig. 4c). The distribution in Fig. 4d shows that among a total of 249 complexes, 98 were bound to nOPI and 151 to nOPII, in agreement with the slightly higher binding affinity of NikR for nOPII observed in footprinting experiments. Volume analysis of the complexes confirms that NikR binds each operator site as a tetramer (see Table 1). In addition, under our imaging conditions, we did not observe DNA looping nor large DNA compaction as it would be expected by the interaction between two NikR tetramers bound to nOPI and nOPII. This result is consistent with the absence of hypersensitive bands in NikR DNaseI footprinting.

### Role of the Fur and NikR operators in *arsR* regulation

To characterize the functional significance of this complex promoter architecture, a bioluminescent *H. pylori lux* reporter strain was used^30^. Wild-type and mutagenized constructs of the P*arsR* promoter were fused upstream of a chromosomal *luxCDABE* reporter cassette and assayed for reporter expression (luminescence) and transcription (quantitative PCR with reverse transcription (RT–qPCR)) shortly after metal repletion or chelation. Concomitantly, Fur and NikR binding to each of these constructs was assayed by DNase footprinting (Supplementary Fig. 4).

**Figure 5:**
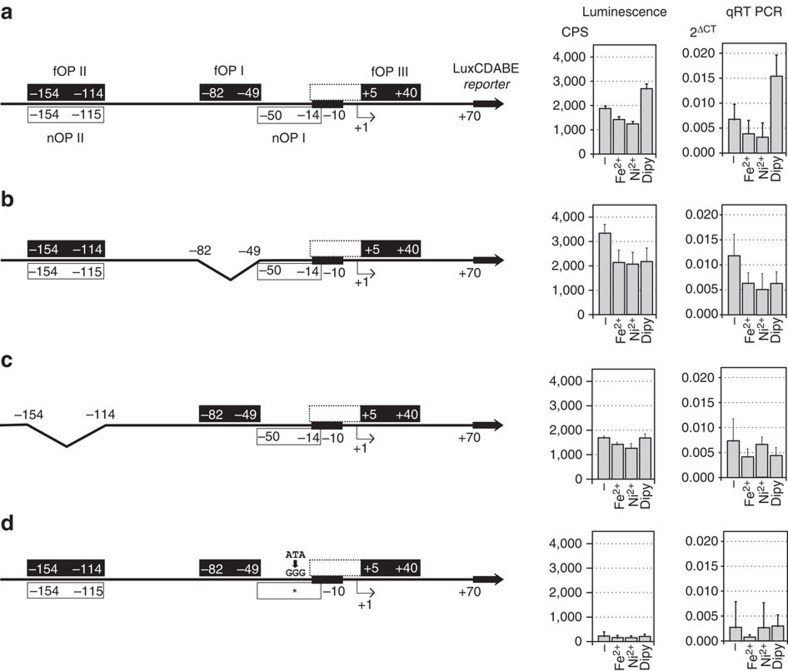
Functional analysis of Fur and NikR operators on P*arsR*. Expression and transcription levels monitored through a P*arsR*–*lux* reporter fusion. Histograms are shown for *in vivo* luminescence and *in vitro* RT–qPCR. (**a**) Wild-type P*arsR* promoter; (**b**) P*arsR* lacking the central holo-Fur operator (ΔfOPI); (**c**) P*arsR* lacking the distal Fur and NikR operators (ΔfOPII/nOPII); (**d**) P*arsR* mutated in the NikR proximal operator nOPI* (ATA→GGG). Fur and NikR operators are depicted as black and white boxes, respectively. From left to right, bars represent the untreated control (−), a 15 min treatment with iron (Fe^2+^), nickel (Ni^2+^) and iron chelator (Dipy). Error marks indicate the standard deviation of eight (luminescence) and four (RT–qPCR) independent experiments.

The luminescence measured from the wild-type P*arsR* promoter fused to the *lux* reporter recall the responses observed in primer extension analysis at the endogenous *ars* promoter (Fig. 1 and Fig. 5a). These responses were independently substantiated also at the transcriptional level by RT–qPCR, indicating that the reporter construct could be reliably used to further dissect the complex operator architecture of P*arsR*.

When the central high affinity holo-Fur operator was deleted, we observed a consistent general de-repression of the promoter with respect to the wild-type construct. Since the fusion still responded to the presence of metals, the de-repression proved to be not constitutive as in the *Δfur* strain (Fig. 5b). In fact, the promoter was consistently repressible by iron and nickel ions with fold-repression levels similar to the wild-type construct, likely due to uncompromised binding of holo-Fur to either fOPII and/or fOPIII (Supplementary Fig. 4B). On the contrary, transcription of the reporter was also inhibited after iron withdrawal, indicating a lack of inducibility.

**Figure 6:**
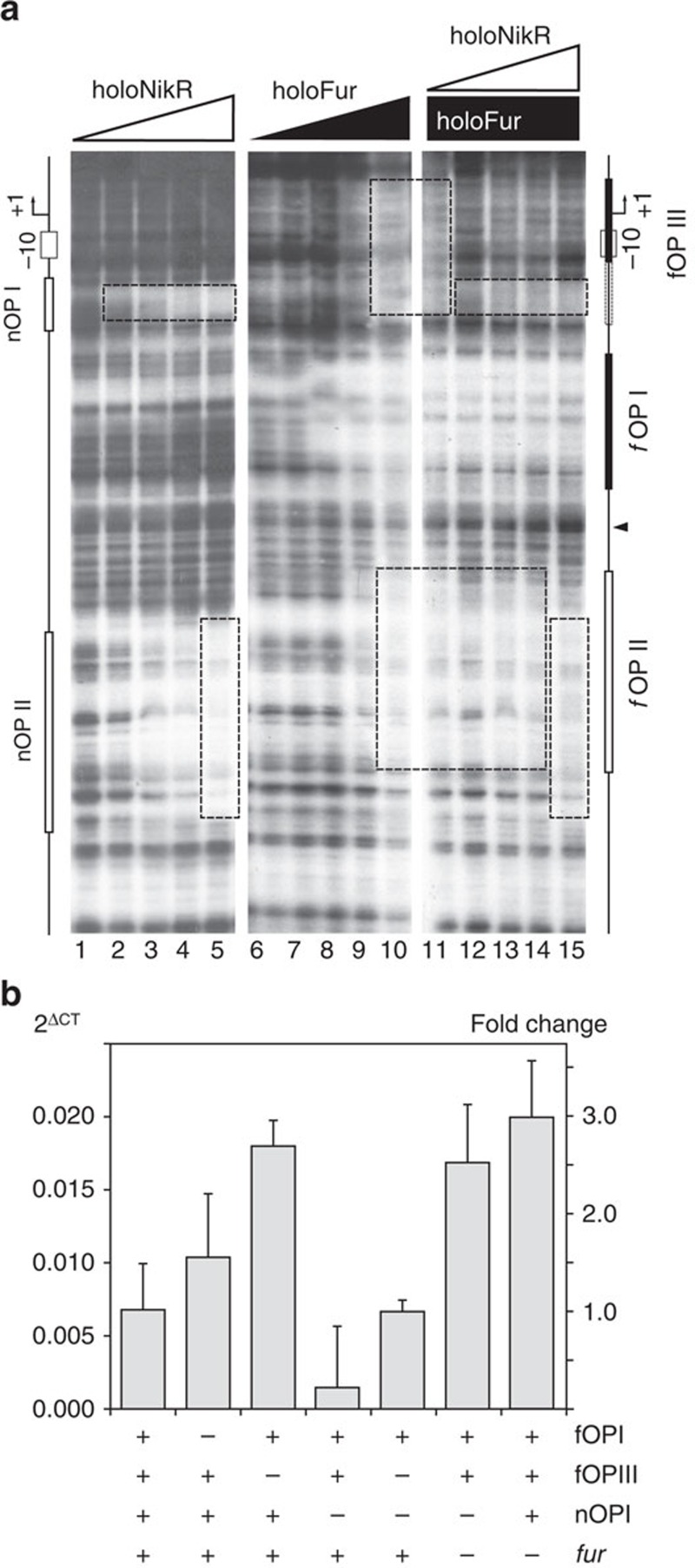
NikR impairs Fur binding to fOPII and fOPIII. (**a**) Competitive footprinting experiment. Lanes 1–5: protection pattern with increasing holo-NikR concentration (0, 4, 8, 16 and 32 nM NikR tetramer, respectively). Lanes 6–10: protection pattern with increasing holo-Fur concentration (0, 4, 8, 17 and 35 nM Fur tetramer, respectively). Lanes 11–15: protection pattern with increasing holo-NikR concentration (as in lanes 1–5) in the presence of 70 nM Fur tetramer. A schematic map of the promoter region is depicted on the right side of the gels. Dashed boxes outline the competitive binding to the distal operator fOPII and impairment of Fur binding to fOPIII upon NikR binding to nOPI. (**b**) Functional analysis of the low-affinity holo-Fur operator (fOPIII) and of the NikR anti-repression element (nOPI). Bars represent the mRNA levels of the P*arsR*–*lux* reporter fusion assayed by RT–qPCR in different genetic backgrounds: wild-type, ΔfOPI, ΔfOPIII, nOPI*, nOPI*-ΔfOPIII, nOPI*-Δ*fur* and Δ*fur* mutants. Error marks indicate the standard deviation of two biological replicates, each analysed twice in independent RT–qPCR runs.

This effect can be explained if the metal-dependent multimerization of Fur is taken into account. In native conditions, with some iron present, Fur will be in dimeric and partly also in tetrameric state (dimer of dimers). Some Fur tetramers may, therefore, bind to fOPII and allow activation, much as the dimer of dimers formed by apo-Fur between fOPI and fOPII appears to induce the promoter (see Fig. 5c below). When metals are in excess, Fur tetramerizes and binds also the fOPIII element, promoting repression (Figs 1, 2, 3, and also Fig. 6b). On the other hand, when iron is chelated by adding dipyridyl, the prevalent state of Fur becomes dimeric, with binding only to fOPII (Supplementary Fig. 4B). However, binding of the apo-Fur dimer *per se* on fOPII seems to be insufficient to allow induction.

Thus, the deletion of fOPI determines a pleiotropic de-repression of the P*ars* promoter and a loss in the response provoked by iron depletion. This suggests that the central high affinity fOPI operator has a dual role: it is crucial for repression—even though dispensable for the transcriptional response to iron (FeOFF)—and, at the same time, it is required for induction of the P*arsR* promoter by apo-Fur. In fact, two concomitant conditions have to be fulfilled to allow Fur-dependent P*arsR* inducibility: the binding of a tetramer or dimer of dimers to a distal (upstream) position and the absence of binding to the downstream fOPIII element (see Fig. 5c below and ΔfOPIII mutant in Fig. 6b, respectively). These data are consistent with the AFM observation indicating that fOPI is involved both in apo- and holo-Fur promoter-DNA compaction.

Next, we deleted the distal operator encompassing the overlapping apo-Fur and NikR binding sites (fOPII/nOPII). Interestingly, this deletion did not impair significantly the basal levels of transcription from P*arsR*, as it did not impair Fur binding to the other operators (Supplementary Fig. 4C). Instead, as in the case of fOPI deletion, we observed a loss of inducibility in response to iron depletion (Fig. 5c). Thus, both fOPI and fOPII are required for Fur-dependent induction of P*arsR*. In light of the apo-Fur mediated DNA compaction observed with AFM, the data indicate that the distal operator fOPII approaches the central operator fOPI through Fur-mediated interactions to ensure P*ars*R induction upon iron chelation. Intriguingly, a similar mechanism has been postulated for the control of the P*fur* promoter itself^31^(see ‘Discussion' section).

To explore the function of nOPI, targeted site-directed mutagenesis of the NikR binding consensus sequence was performed, substituting three critical nucleotides of the hemi-operator motif (ATA→GGG)^14^, while leaving the −10 box intact (nOPI*, Fig. 5d). Footprinting analysis confirmed that binding of NikR to this mutated element was abolished (Supplementary Fig. 4D), with a consequent drastic reduction of the transcript levels (Fig. 5d). Nevertheless, the transcriptional response resulting in iron-dependent repression (FeOFF) was maintained, reproducing to a large extent the responses observed at the endogenous P*ars* promoter in the Δ*nikR* strain (see Fig. 1). These results indicate that NikR can either activate the *ars* promoter directly, as a class I activator, or indirectly through an anti-repression mechanism that impairs the binding of Fur to the *arsR* promoter. However, although some MerR-type activators have been reported to activate transcription by binding and changing the conformation of a nonoptimal spacer sequence between −10 and −35 boxes^32^, the position of the nOPI operator would be rather unconventional for a RHH-regulator-like NikR to act as such a transcriptional activator. Accordingly, the primer extension analyses clearly show that in the double mutant background (Δ*fur*Δ*nikR*), the endogenous P*ars* promoter is constitutively de-repressed (Fig. 1). This is a strong indication that NikR may act as an anti-repressor of the Fur-regulated *ars* promoter, by binding to nOPI.

### NikR has an antagonistic effect on Fur binding and repression

To further characterize the regulatory interplay between Fur and NikR, we performed competitive footprinting experiments by varying the concentrations of one regulator in the presence of a fixed amount of the second, and vice versa. Except for the distal overlapping fOPII/nOPII operator, for which both proteins competed as expected, increasing amounts of Fur had no effect on the binding affinity of NikR to nOPI, indicating absence of positive or negative cooperativity at this operator (data not shown). Conversely, when increasing amounts of NikR were added to the preformed Fur–DNA complexes, we observed a marked reduction of the Fur footprint at the low-affinity fOPIII operator, together with the anticipated Fur-NikR competition for the distal fOPII site. Interestingly, protection of the high-affinity operator fOPI remained unaltered (Fig. 6a).

These results further support the hypothesis that binding of NikR to the *ars* promoter impairs binding of Fur to fOPII and to the fOPIII low-affinity operator, most probably by destabilizing the repressive P*arsR* DNA compaction revealed by AFM, in agreement with the putative anti-repressor function of NikR. To validate this hypothesis, we quantified by RT–qPCR the transcript levels of the P*arsR lux* reporter constructs mutated in the principal Fur and NikR operators (fOPI and nOPI) and in the low-affinity fOPIII Fur operator, in different genetic backgrounds (Fig. 6b). In a wild-type background, deletion of fOPI resulted in de-repression of P*arsR,* while a stronger de-repression of the promoter was observed in the Δ*fur* background or when the fOPIII operator was deleted. On the contrary, mutagenesis of the proximal NikR binding site (ATA→GGG, nOPI*) determined a substantial downregulation of P*ars*. When the latter construct was analysed in a Δ*fur* genetic background, the transcript levels increased more than 10-fold, exceeding the levels observed in the case of fOPI deletion. Thus, the strong downregulation observed after nOPI disruption is a consequence of Fur hyper-repression rather than loss of NikR activation. Finally, when the nOPI* mutation was combined with a deletion of the low affinity fOPIII Fur operator, Fur hyper-repression was relieved, partially reproducing the results obtained for nOPI* in the Δ*fur* strain. Overall, these data demonstrate that fOPIII-mediated promoter compaction is a key element for the repression of *arsR* transcription and that the anti-repressive effect of NikR arises from the antagonistic effect on Fur binding to fOPII and fOPIII. These data also show that the three Fur operators synergistically contribute to P*arsR* repression, since deletion of either fOPI, fOPII or fOPIII fails to de-repress P*arsR* at the same levels observed in the Δ*fur* knockout strain. This supports the evidence of a repressive holo-Fur-dependent DNA compaction of the *arsR* promoter, which can be unwrapped by holo-NikR binding.

## Discussion

Complex promoter architectures, allowing binding of multiple regulators to different operators, provide layers of integration for coordinated transcriptional responses to diverse environmental stimuli. Although to date, relatively few examples have been experimentally investigated in mechanistic detail, DNA looping or bending was frequently shown to be a common hallmark. The GalR-HU repressosome or the CAP-antagonized AraC-looping represent prototypical examples^33^,^34^. Several advantages have been associated with DNA bending, looping and compaction, including a higher local concentration of the repressor^3^,^35^, fast switching between repressed and de-repressed states due to prompt recapture of the regulator by the main operator^6^, attenuation of stochastic fluctuations and transcriptional bursts^36^, faster recognition of target sites^37^ and even bistability^38^.

**Figure 7:**
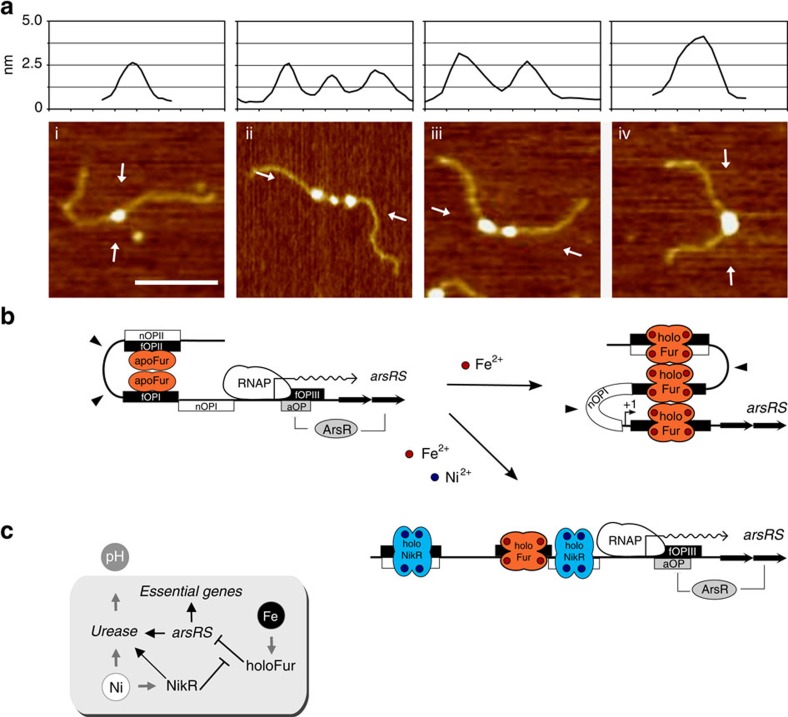
P*arsR* regulation model. Scheme of P*arsR* regulation. (**a**) AFM images of different holo-Fur/P*arsR* pseudo-knotting intermediates. The evident shortening of the DNA fragment and the concomitant enlargement of the nucleo-protein complex are indicative of a significant DNA condensation of the *arsR* promoter region and Fur oligomerization. (**b**) Proposed FeOFF-NikRON model for P*arsR* transcription regulation. At low iron concentration, apo-Fur forms dimers that bind to the upstream operators fOPI and fOPII, looping the intervening DNA but leaving the promoter elements open for RNAP binding and transcription. At high iron concentration, holo-Fur forms tetramers and higher order oligomers that condense the promoter DNA by binding to the three operator sites fOPI, fOPII and fOPIII, thus occluding RNAP binding (FeOFF). In the presence of nickel, holo-NikR binds to the operators nOPI and nOPII preventing Fur binding and promoter condensation, a condition that favours RNAP binding and *arsR* transcription (NikRON). An additional layer of control is introduced by the previously reported feedback regulation mechanism of *arsR* transcription. (**c**) Coherence of metal-dependent *arsRS* regulation with *H. pylori* acid acclimation needs: NikR-dependent antirepression of P*arsR* is possible only when the intracellular nickel levels are sufficiently high to cofactor the urease nickel-enzyme.

Here, we provide the first evidence that a member of the Fur regulators family is capable of inducing different DNA compaction according to the metallation state of the protein. In particular, on iron withdrawal, Fur forms dimers that bind the operators in the upstream promoter region by condensing the intervening DNA, thus inducing *arsR* transcription under iron-limiting conditions. Conversely, in the presence of iron, promoter compaction is achieved through the formation of a nucleoprotein complex involving three holo-Fur tetramers bound to three distinct operators. This mechanism envisages sequential binding events of the repressor as captured by AFM (Fig. 7a). The operators appear to be cooperatively involved in the repression of the *arsR* promoter, much alike the three operators of the Lac operon^39^. The observation that single deletions of fOPI, fOPII and fOPIII fail to de-repress the promoter at the same levels observed in the Δ*fur*-knockout strain (Figs 5 and 6), strengthen this interpretation. Thus, although the Fur central operator fOPI does not overlap the core promoter elements as often observed for canonical repressors, the bidentate nature of the *arsR* promoter readily contributes to enhance repression^5^. Bidentate promoter architectures have been shown to increase the effective repressor concentration at the principal operator by binding the repressor to auxiliary operators and exploiting DNA looping^40^. Notably, our results indicate that this mechanism may be conserved in the Fur repressor family, since these regulators form higher order oligomers and bind multiple operators in many bacterial species (see Introduction).

A particularly interesting aspect of these findings is that different metal ions (Ni^2+^ and Fe^2+^) appear to affect transcription positively or negatively by modulating DNA compaction, according to the metallation state of the two regulators (Fig. 7b). Although the precise DNA path within the Fur nucleoprotein complex cannot be resolved by AFM, thus precluding the possibility to discriminate between looping and wrapping, functionally similar mechanisms have been observed at several DNA looping-prone promoters, demonstrating both negative and positive control according to the presence of different effectors modulating DNA topology^2^. In particular, the anti-repressive effect of NikR observed on the Fur-dependent compaction of the P*arsR* promoter, functionally recalls the disruptive effect of CRP on AraC-mediated loop formation at the *ara* operon^33^. Thus, the specific and opposite transduction of cognate stimuli (specific metal ions or distinct sugars) appears to be similarly modulated by a balance of DNA condensation and decondensation mechanisms, mediated by different effectors. Interestingly, the presence of an antagonistic operator, promoting the disruption of the interaction between the main and auxiliary operators of the main regulator, seems to be a common theme in the architecture of these complex promoters (for example, CRP versus araI1 and araO2 in P*BAD*, CRP versus O1 and O3 in P*lac* and nOPI versus fOPI and fOPIII in P*arsR*) (Supplementary Fig. 5). This suggests promoter compaction and relaxation as an evolutionary conserved mechanism to integrate transcriptional responses at bacterial promoters.

Remarkably, this complex-promoter architecture, made of overlapping and adjacent Fur and NikR cis elements, is found at several metal-responsive promoters in *H. pylori*. For example, the architecture of the *fur* promoter itself structurally and functionally resembles that of the *arsR* promoter (Supplementary Fig. 5)^31^. Likewise, the divergent P*exbB*–P*nikR* promoter region encompasses three Fur operators, including a central element^14^. Notably, in both P*fur* and P*exbB*–P*nikR*, the NikR operators match the positions mapped on the P*arsR* promoter, with a distal NikR element overlapping the upstream auxiliary Fur operator and a second proximal NikR element located between the central and the downstream auxiliary Fur operators (Supplementary Fig. 5). These similarities suggest that promoter compaction and anti-repression mechanisms may be adopted in the regulation of other operons where an integrated response to iron and nickel is required.

The tight transcriptional integration between these two metal ions is not surprising, since they may compete for the same uptake and storage proteins, activate similar redox detoxifications systems and, in some cases, participate together as cofactors of key metallo-enzymes (for example, Ni–Fe hydrogenase)^41^. In this respect, our results contribute to unify acid-acclimation and metal-dependent responses in a mechanistically and physiologically coherent model. Specifically, the acid-inducible and ArsR-dependent expression of the *H. pylori* urease cistrons is supported only when the intracellular Ni^2+^ levels are sufficiently high to cofactor NikR and the urease holo-enzyme (NikRON) and iron levels are not too high to cause redox toxicity (Fig. 7c). At low nickel concentration, not only is the expression of urease not useful, but also other vital functions dependent on the essential gene targets of ArsR need to be downregulated since the growth in the acid gastric niche is possible only under the pH-buffering conditions provided by urease. Similarly, if the intracellular concentration of iron is too high, *arsR* transcription, and thereby the expression of essential regulatory targets and ultimately growth, will be downregulated even in the presence of nickel (FeOFF), to avoid redox stress, consistent with the microaerophilic metabolism of *H. pylori* and the involvement of Fur in redox regulation^11^,^42^. Finally, P*arsR* is also controlled through the apparently negative auto-regulation by ArsR^26^, introducing an additional layer of feedback regulation in ArsR homeostasis.

This signal integration logic well fits the pathobiontic nature of *H. pylori*, balancing metabolic needs and stealth behaviour to avoid host responses and counter acidic as well as oxidative stresses.

In conclusion, our results provide for the first time evidence of a metal-dependent mechanism of DNA compaction mediated by a member of the widespread Fur family of metallo-regulators (Fig. 7b). This mechanism feeds directly into the control of *H. pylori* acid acclimation and growth, through short-range DNA interactions of the *arsR* promoter, antagonized by NikR. Together with DPS, a nucleoid-associated protein that mediates the pH-dependent DNA condensation in *H. pylori*^43^, Fur provides the capability to condense DNA in a metal-dependent fashion, a feature that may be also relevant for the formation of *H. pylori* coccoid forms, especially in the late stationary phase, when Fur concentration has been reported to increase^19^.

More in general, we notice that together with the DNA-shaping features reported in this study, many Fur orthologues appear to recapitulate features characteristic of NAPs and global regulators such as elevated number of intracellular copies, the formation of higher order multimers, promiscuous binding specificities, including DNA shape readout and minor groove readout mechanisms^12^,^44^, as well as elevated numbers of genomic binding sites, even at 'orphan' positions, which map distantly from TSSs^19^,^45^,^46^.

If transcriptional regulators evolved from nucleoid-associated proteins^47^, it is striking to notice that Fur proteins are conserved also in bacteria that have undergone reductive evolution, finally encoding only few classical transcription factors, and a reduced cohort of nucleoid-associated proteins (for example, *Mycoplasma*^48^, *Bacteroides*^49^, and so on). Therefore, it is not unrealistic to hypothesize that many Fur orthologues may play the double role of specific metallo-regulators as well as structural mediators of the nucleoid organization through DNA looping or wrapping interactions.

## Methods

### Bacterial strains and culture conditions

*H. pylori* strains (Supplementary Table 1), were revitalized from glycerol stocks on Brucella broth agar plates added with 5% fetal calf serum and 0.2% cyclohexamide and Dent's antibiotic supplement under microaerophilic conditions (Oxoid) for 2 days. After re-streaking on fresh plates, bacteria were cultured in a 9% CO_2_–91% air atmosphere at 37 °C and 95% humidity in a water-jacketed incubator (Thermo Forma Scientific). Liquid cultures were grown in modified Brucella broth medium supplemented with 5% fetal calf serum, 0.2% cyclohexamide and Dent's antibiotic supplement in glass flasks or 25 cm^3^ sterile plastic flasks with vented cap (Corning).

### Primer extension analysis

Primer extension analysis on the native P*arsR* promoter was performed using oligo 166 pe3 (Supplementary Table 2), as previously described in ref. 50.

### P*arsR-lux* reporter strains

Bioluminescent P*arsR* reporter strains were generated by natural transformation of a G27 *lux* acceptor strain^30^. In particular, the promoterless *luxCDABE* reporter was put under the control of the wild-type or mutant forms of the *arsR* promoter by double homologous recombination following transformation with 5 μg of a pVCC suicide vector (Supplementary Table 3); positive clones were selected on Brucella broth agar plates supplemented with chloramphenicol at 30 μg ml^−1^. The P*arsR* wild-type promoter was amplified from genomic DNA by PCR using oligonucleotides 166 pe3/166*Alida* (Supplementary Table 2). The amplicon was cloned into a pGEMT-Easy TA-cloning vector (Promega). From this plasmid, the promoter sequence was excised with NcoI/NdeI digestion, made blunt-ended with Klenow fragment and ligated to the blunt-ended BamHI site of pVCC (Supplementary Table 3). The mutant promoters were obtained as synthetic genes from GENEWIZ (Supplementary Table 4), subcloned into the BamHI site of pVCC and validated by sequencing. The constructs were used to transform the G27 *lux* acceptor strain (Supplementary Table 1). To assess bioluminscence, 20 ml of pre-heated Brucella broth medium were inoculated with an overnight culture of the desired strain (starting *D*_600_ 0.07) and grown until early exponential phase. The culture was divided into four 1-ml aliquots in a 24-well tissue culture plate (Corning) and treated for 20 min with 1 mM (NH_4_)_2_Fe(SO_4_)_2_, 1 mM NiSO_4_, or 150 μM 2-2 dipyridyl. Luminescence emission was monitored every 20 min with a EnSpire Multiplate reader (PerkinElmer). For transcriptional analysis with RT–qPCR, a 20 ml culture obtained as described was divided into four 5 ml aliquots and treated with the same metals or chelators before RNA extraction, cDNA synthesis with the oligo luxC3, and qPCR with primers LuxRT FW/RV (Supplementary Table 2), all according to ref. 42.

### DNAse I footprinting

NikR and Fur recombinant proteins were purified as previously described^27^,^50^. For probe preparation, plasmids carrying either the wild-type or the mutant P*arsR* promoter (Supplementary Table 3) were linearized with NcoI or BamHI, 5' end labelled with [γ-^32^P]-ATP by T4 polynucleotide kinase and gel purified after a second cut at the 3' end; 15 fmol of labelled probe were used for each footprinting reaction. The footprinting assays with Fur alone were performed in 1X Fur Footprinting Buffer (FPB) according to ref. 42, incubating different amounts of protein for 20 min at room temperature with 300 ng of nonspecific competitor salmon sperm DNA, 150 μM (NH_4_)_2_Fe(SO_4_)_2_ or 150 μM 2-2 dipyridyl and 15 fmol of labelled DNA probe in a final volume of 50 μl. Footprinting assays with NikR were performed similarly, in 1X NikR FPB according to ref. 27. NikR was pre-equilibrated in 1X NikR FPB overnight at 4 °C, in a final volume of 10 μl. Footprinting assays performed with both Fur and NikR were made in 1X Competitive FPB (20 mM HEPES pH 7.85; 50 mM KCl; 0.02% Igepal; 0.4 dithiothreitol (DTT), 10% glycerol), containing 300 ng of sonicated salmon sperm DNA, 150 μM of (NH_4_)_2_Fe(SO_4_)_2_ or 150 μM 2-2 dipyridyl and 15 fmol of labelled DNA probe. Fur was pre-equilibrated in 1X Fur FPB with 5 mM DTT for 15 min before addition to the mix. After 10 min of probe incubation with Fur at room temperature, NikR and 150 μM NiSO_4_ were added, and the reaction was incubated for additional 10 min before DNA digestion. The partial digestion of the labelled probes was carried out in the presence of 10 mM CaCl_2_ and 5 mM MgCl_2_, varying DNase I concentration (0.05–0.3 U; Novagen) and incubation times (60–75 s), to reach comparable digestion ladders in sequencing gels.

### Sample preparation for AFM imaging

The five globular proteins used to obtain the calibration curve relating AFM volume and molecular weight were: *Equus caballus* myoglobin (17 kDa), Bovine pancreas DNase I (30 kDa), Bovine serum albumin (67 kDa), Bovine liver catalase (250 kDa) and *Escherichia coli* RNA polymerase-σ^70^ (458 kDa; Supplementary Fig. 1). Apo-Fur and holo-Fur were prepared by incubating the protein with 150 μM of 2,2'-dipyridyl or 150 μM (NH_4_)_2_Fe(SO_4_)_2_·6H_2_O, respectively, in binding buffer (25 mM sodium phosphate buffer pH 8, 70 mM NaCl, 15 mM KCl, 0.1 mM DTT) at room temperature for 15 min. To avoid subunits dissociation upon deposition, the protein was crosslinked with 10 mM glutaraldehyde for 2 min. The crosslinking reaction was quenched with 1 M Tris-HCl pH 8 (final concentration of 60 mM). Each protein was diluted in deposition buffer to a final concentration of 5–50 nM and deposited onto freshly cleaved mica as described above.

The 818 bp-long DNA template harbouring the P*arsR* promoter was obtained by PCR from plasmid pGEM-P*arsR* using Taq DNA polymerase in standard reaction conditions. pGEM-P*arsR* was constructed by cloning the P*arsR* promoter region (from position −203 to +61 with respect to TSS) of *H. pylori* G27 into the poly-linker of plasmid pGEM-T easy (Promega; Supplementary Table 3). The DNA fragment was gel purified by electroelution using an Elutrap apparatus (Schleicher & Schuell, Keene NH), phenol/chloroform extracted, ethanol precipitated and resuspended in 5 mM Tris-HCl pH 7.4. The DNA concentration was determined by absorbance at 260 nm.

The 1,129 bp-long DNA template used in experiments shown in Supplementary Fig. 3 was obtained by PCR using primers P*arsR*_For and P*arsR*_Rev (Supplementary Table 2) and plasmid pGEM-P*arsR*2 as template (Supplementary Table 3). pGEM-P*arsR*2 was obtained from pGEM-P*arsR* in two steps. First, a BamH1 restriction site was inserted between the fOPI and fOPII sites by mutagenic PCR using primers P*arsR*mut_For and P*arsR*mut_Rev (Supplementary Table 2). Next, a 315 bp DNA insert obtained by PCR from pET28b plasmid using primers P*arsR*ins_For and P*arsR*ins_Rev (Supplementary Table 2) was cloned into the created BamH1 restriction site. The DNA fragment was purified as described above.

### Complexes assembly for AFM imaging

Fur–DNA complexes were assembled in 1X Fur footprinting buffer containing either 150 μM 2,2'-dipyridyl (apoFur) or 150 μM (NH_4_)_2_Fe(SO_4_)_2_·6H_2_O (holo-Fur). The 10 μl reaction containing 9 nM DNA and 380 nM Fur dimers was incubated for 15 min at 25 °C. Afterwards, glutaraldehyde at a final concentration of 10 mM was added to the reactions, followed by an incubation of 2 min at room temperature. The crosslinking of the Fur-binding reaction with glutaraldehyde was introduced because Fur oligomers had the tendency to disrupt upon deposition onto mica (Supplementary Fig. 2). The crosslinking reaction was quenched with 1 M Tris-HCl pH 8 (final concentration 60 mM). Two microlitres of the reaction were diluted in 20 μl of deposition buffer (4 mM HEPES pH 7.4, 10 mM NaCl, 2 mM MgCl_2_) and immediately deposited onto freshly cleaved mica. After 3 min, the mica disk was rinsed with milliQ water and dried with a gentle stream of nitrogen. AFM analysis of the binding of NikR to the P*arsR* promoter was more straightforward because holo-NikR forms stable tetramers, which are not disrupted upon deposition onto mica and do not form oligomers of high molecular mass. NikR–DNA complexes were assembled in 1X NikR FPB (20 mM HEPES pH 8, 50 mM KCl, 1 mM NiSO_4_, 0.1 mM DTT). The 10 μl reaction containing 10 nM DNA and 640 nM NikR tetramers was incubated for 15 min at 25 °C. The reaction was diluted 1:10 in deposition buffer and deposited onto freshly cleaved mica for 3 min. Afterwards, the mica disk was rinsed with milliQ water and dried with nitrogen.. AFM imaging was carried out with tapping mode in air with a Nanoscope IIIA (Digital Instruments, Santa Barbara, CA, USA) microscope equipped with a 12 μm scanner (E scanner) and commercial silicon cantilevers (MikroMasch, Tallinn, Estonia). Square images of 512 × 512 pixels were collected with a scan size of 1 μm at a scan rate of two lines per second.

### AFM image analysis

Images were analysed using locally written Matlab scripts and Gwyddion software (v. 2.37). DNA contour length measurements were performed by manually tracing the DNA contour from one end to the other. The digitized DNA trace served as an outline to identify the subset of pixels with higher intensity of the DNA backbone. Images were skeletonized with the *bimorph* built-in function of Matlab to generate eight connected chaincode of unit thickness and the DNA contour length was determined by (*n*_e_*,n*_o_)-characterization using the following contour length estimator: PAGE \*Arabic 21 *L*_DNA_=(0.963 *n*_e_+1.362 *n*_o_) × *S*/*W*, where: *n*_e_ and *n*_o_ are the number of even- and odd-chain pixel, respectively, *S* is the image scan size (1,000 nm), *W* is the image width (512 pixels; ref. 51). Because the DNA path in proximity of the protein cannot be seen, the DNA trace was made to pass through the centre of the protein. Short distances between Fur oligomers bound to the different operators were measured with the straight line measuring tool of the Nanoscope software. The position of Fur oligomers or NikR tetramers bound along the DNA template was selected with the mouse by clicking in the centre of each globular feature.

To assess the stoichiometry of these oligomers, we constructed a molecular mass–volume calibration curve using a set of globular proteins of known molecular mass (Supplementary Fig. 1). Protein volume analysis of molecular weight markers, apo-Fur and holo-Fur in the absence of DNA were measured using the thresholding algorithm of the Gwyddion particle analysis procedure. Volume of Fur oligomeric states and NikR tetramers bound to the different operator sites were measured by single grain analysis using Matlab and *ad hoc* scripts. After defining the particle boundaries with the free hand tool, the mean height of the boundary pixels was determined and used as reference background. The volume of the particle was then computed by multiplying the area of the particle boundary by the average pixel height, relative to the reference background, of the particle.

Notice that for small proteins, the DNA contributes significantly to the volume of the complex and, therefore, it must be taken into account when inferring its molecular mass. A small DNA compaction is generally due to the different path that the DNA can take upon deposition. Namely, if the complex sticks to the surface in an orientation such that DNA has to detach from the surface to overcome the protein, its DNA contour length will be reduced by a few nanometers depending on the size of the protein. As the volume of the complexes increases, the uncertainty of the volume measurements becomes larger. This is, in part, due to the broadening effect of the AFM tip, which is larger for bigger objects and in part due to the fact that the density of the complex oligomeric nucleo-protein aggregates may be lower than that of the globular proteins used as standards.

### Data availability

The data that support the findings of this study, as well as the *ad hoc* scripts for AFM image analysis, are available from the corresponding author upon request.

## Additional information

**How to cite this article:** Roncarati, D. *et al*. Metal-responsive promoter DNA compaction by the ferric uptake regulator. *Nat. Commun.* 7:12593 doi: 10.1038/ncomms12593 (2016).

## Supplementary Material

Supplementary Information Supplementary Figures 1-6, Supplementary Tables 1-4 and Supplementary References

## Figures and Tables

**Table 1:** AFM measurements of Fur and NikR nucleoprotein complexes with P*arsR*.

	**Volume (nm**^**3**^)	**Inferred MW (kDa)**	**DNA compaction (nm)**	**Oligomerization state**
Apo-Fur	123±33	56±21	0.0±2.0	One dimer
Apo-Fur	270±56	113±30	17.4±2.5	Two dimers
Holo-Fur	220±39	93±24	7.4±3.0	One tetramer
Holo-Fur	568±136	228±61	14.9±1.8	Two tetramers
Holo-Fur	1089±244	430±103	65.4±10.8	Three or more tetramers
Holo-NikR-nOPI	257±36	102±14	11.8±1.3	One tetramer
Holo-NikR-nOPII	243±37	96±12	10.8±1.1	One tetramer
