# Supplementary material for: Metal-responsive promoter DNA compaction by the ferric uptake regulator
Source: Nat Commun. 2016 Aug 25;7:12593. doi: 10.1038/ncomms12593 (PMC5007355; doi:10.1038/ncomms12593)
Supplement: Supplementary Information — Supplementary Figures 1-6, Supplementary Tables 1-4 and Supplementary References [file ncomms12593-s1.pdf]

Metal-responsive promoter DNA compaction by the Ferric Uptake Regulator

Roncarati, Pelliciari, Doniselli et al.

Supplementary Information

SUPPLEMENTARY FIGURES

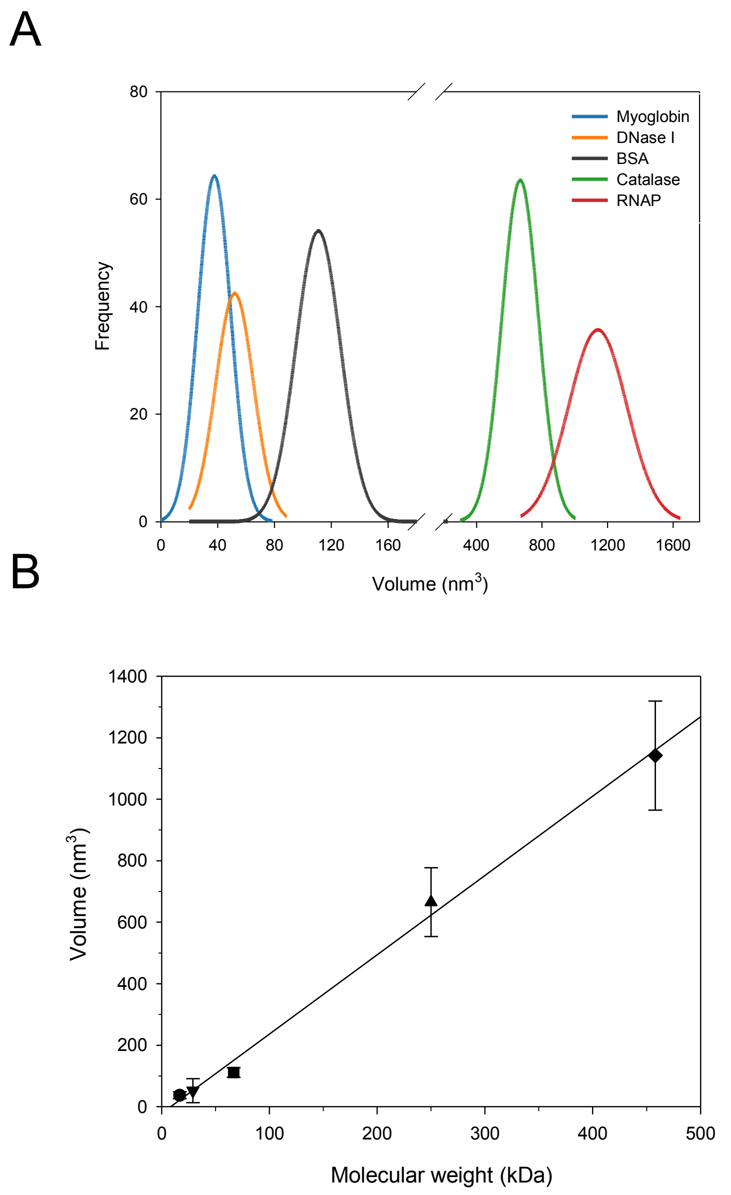

Supplementary Figure 1. Molecular mass calibration curve. A) Volume distributions determined by AFM of five globular proteins with known molecular mass. From left to right: Myoglobin (17 kDa), DNase I (29 kDa), BSA (67 kDa), Catalase (250 kDa) and *E. coli* RNA polymerase-σ^{70} (458 kDa). B) The mean of each volume distribution is plotted against the molecular mass of the protein. The error bars are the standard deviation of the distributions shown in panel A.

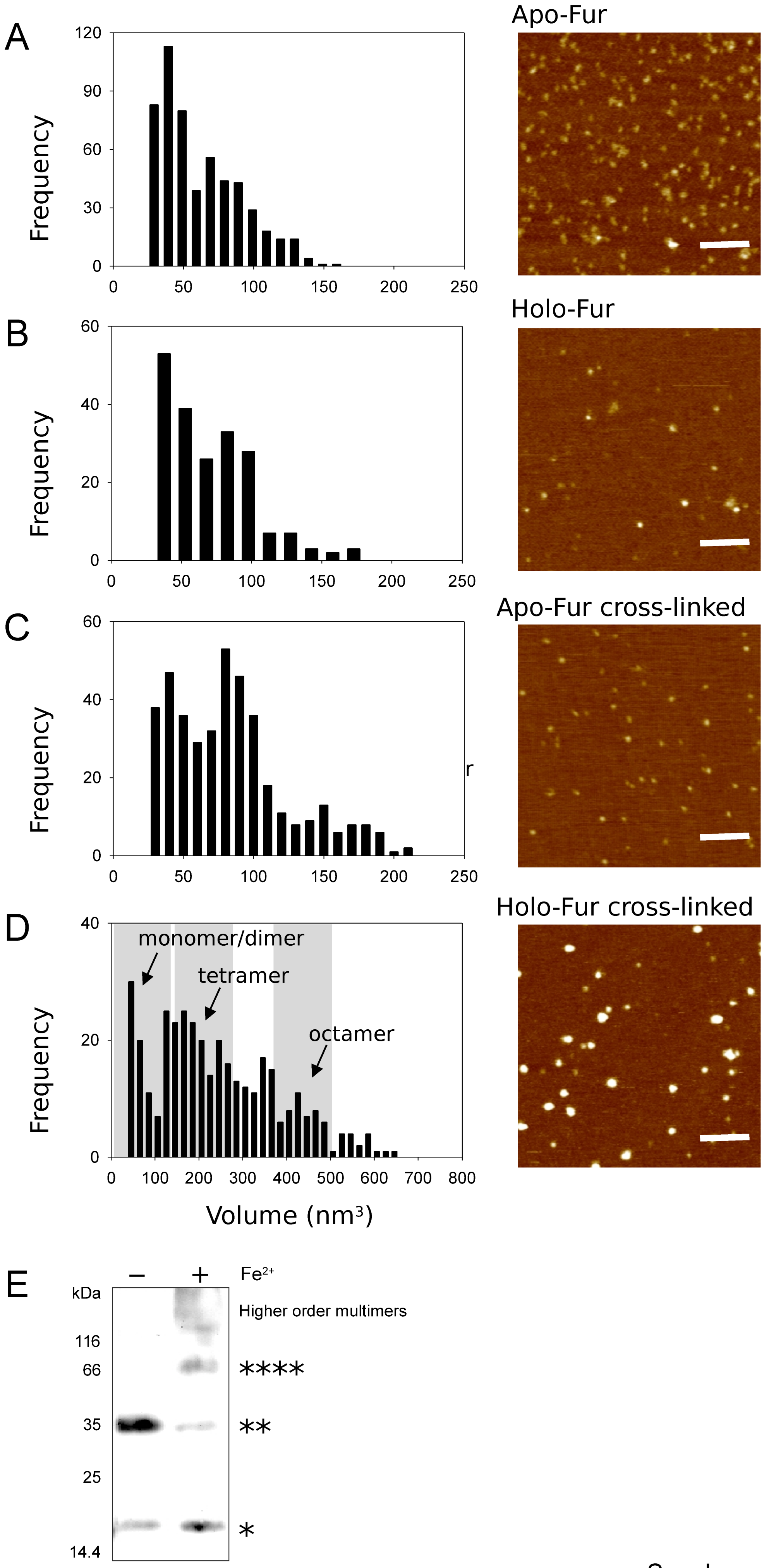

Supplementary Figure 2

Supplementary Figure 2. AFM volume measurements and immunoblot of different Fur oligomeric states. A) Apo-Fur; B) Holo-Fur; C) Apo-Fur cross-linked with glutaraldehyde; D) Holo-Fur cross-linked with glutaraldehyde. A representative AFM image of each Fur state is shown on the right side. The bin size is 10 nm^3 for histograms in A, B and C and 20 nm^3 for histogram in D. Image bar 100 nm. E) Immunoblot of apo- and holo-Fur cross-linked with glutaraldehyde under the same conditions used for AFM measurements. Immuno blotting was performed as described in [1]. Asterisks denote the resulting oligomeric state of Fur (*, monomer; **, dimer; ****, tetramer), desumed from a protein molecular weight marker run in parallel (Pierce).

Single particle volume analysis of apo- and holo-Fur reveals the presence of two populations of molecules, one with a volume of ~40 nm^3 and the other with a volume of ~80 nm^3, suggesting that under the conditions used for AFM imaging apo- and holo-Fur exists as monomers and dimers (Supplementary Fig. 2A and 2B).
Because the interaction with the highly hydrophilic mica surface can potentially alter the oligomeric state of proteins, apo- and holo-Fur were subjected to glutaraldehyde crosslinking for 2 minutes before deposition. Supplementary Fig. 2C shows that under these conditions, and similarly to what observed without crosslinking, apo-Fur is mostly comprised of monomers and dimers even though the dimeric species is more populated than the monomeric one and a discrete number of particles have a volume of ~150 nm^3 (about four times that of the monomer) which may represent Fur tetramers. Conversely, the results obtained with crosslinked holo-Fur are remarkably different (Supplementary Fig. 2D). In particular, we observe a wide distribution of protein particles with volumes up to 600 nm^3. To assess the stoichiometry of these oligomers we constructed a molecular mass-volume calibration curve using a set of globular proteins of known molecular mass (Supplementary Fig. 1B). From this calibration curve we could infer that the oligomers formed by holo-Fur comprise dimers, tetramers, octamers and even higher oligomeric states. This analysis also suggests that glutaraldehyde crosslinking is required to prevent subunit dissociation induced by the interaction with the mica surface. The broad distribution of volumes may be due to the incomplete crosslinking of the subunits and to the different orientation of the oligomers on the surface.

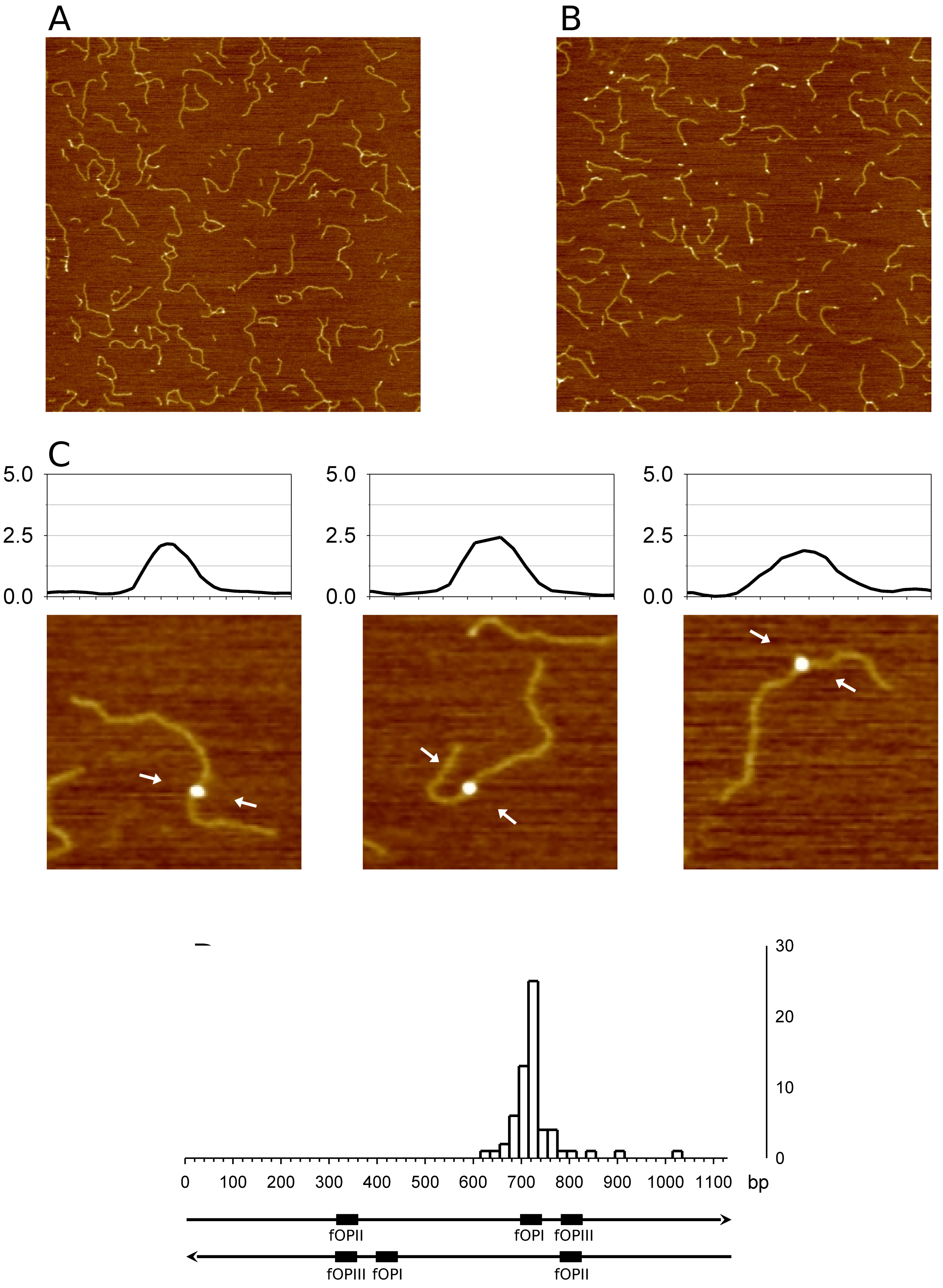

Supplementary Figure 3

Supplementary Figure 3. **Crosslinking experiments with DNA alone and Holo-Fur complexes on mutated P*arsR*.** AFM images of 818 bp DNA fragments crosslinked with glutaraldehyde at a final concentration of 10 mM for two (A) and ten (B) minutes. No significant DNA distortion or compaction was observed. C) Selected Holo-Fur DNA complexes assembled onto the 1129 bp long P*arsR* promoter mutated with the insertion of a 315 bp spacer between the fOPI and fOPII sites. With this DNA template we did not observe neither the large globular features found with the wild-type promoter under the same experimental conditions and shown in Fig. 3F, nor DNA looping mediated by the interaction of holo-Fur bound to fOPI and fOPII. The image profile along the direction indicated by the white arrows is very similar to that observed in Fig. 3D, thus suggesting that the observed complexes correspond to one holo-Fur tetramer. D) Bar chart representing the position of holo-Fur bound along the DNA template (black arrow) with the fOPI, fOPII and fOPIII sites represented, in scale, as black boxes. Because we cannot distinguish the two DNA ends, the DNA templates is shown in the two possible orientations. As expected, the majority of the complexes are formed on the fOPI site. Graph scale in base pairs.

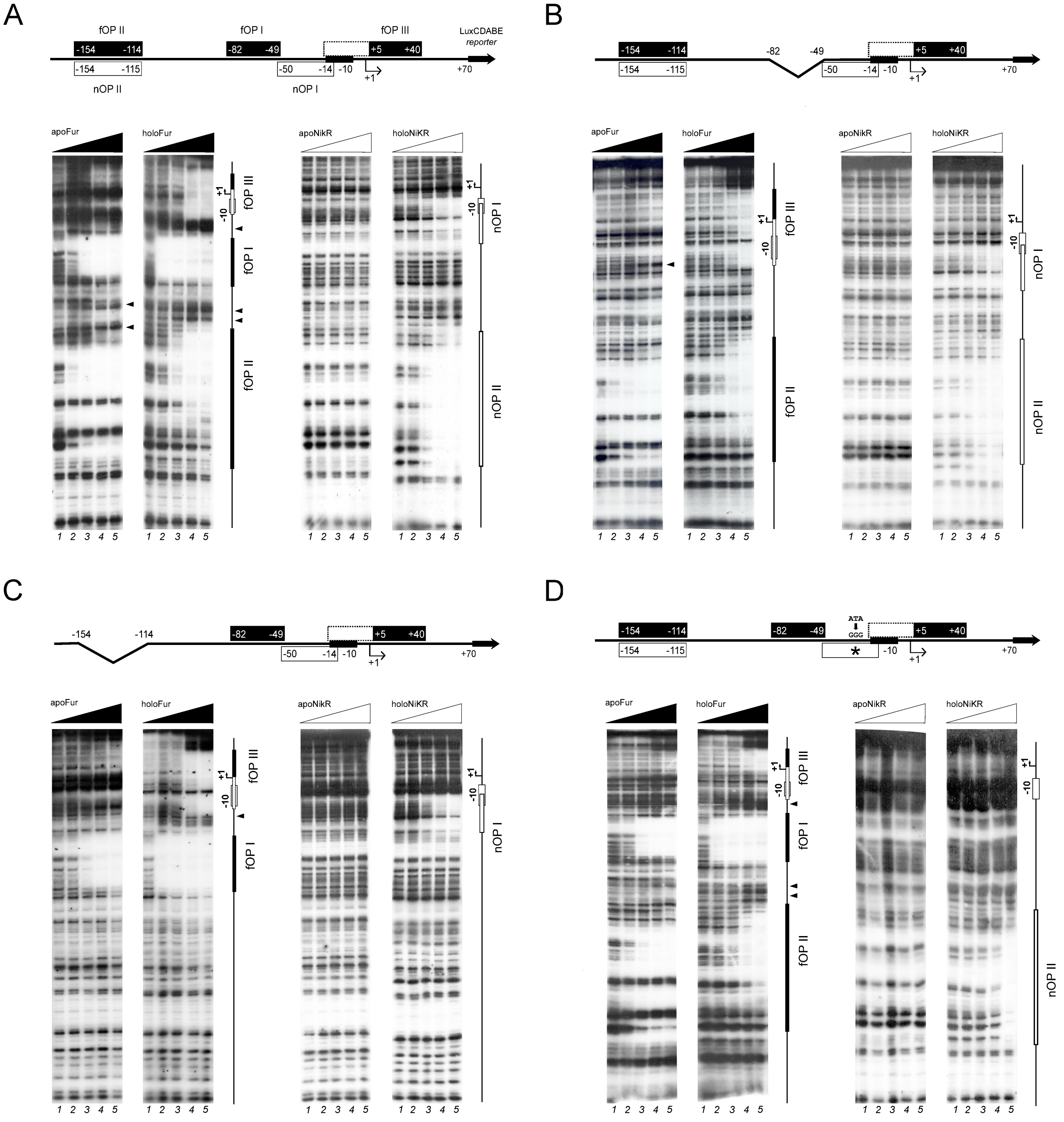

Supplementary Figure 4. Fur and NikR DNase I footprinting on wild-type and mutant P*arsR* promoter constructs. Fur and NikR operators are shaded in black and in white, respectively. Arrowheads indicate bands of hypersensitivity. Numbers refer to the position with respect to the TSS. Lanes 1-5: 0, 17, 35, 70 and 140 nM of either apo- and holo-Fur dimers (left panels) or apo- and holo-NikR dimers (right panels). A) Wild-type P*arsR* promoter probe; B) Mutant promoter lacking the central Fur operator fOPI; C) P*arsR* mutant lacking the distal operators fOPII and nOPII; D) P*arsR* mutant with the ATA → GGG substitution in the proximal NikR operator (nOPI*).

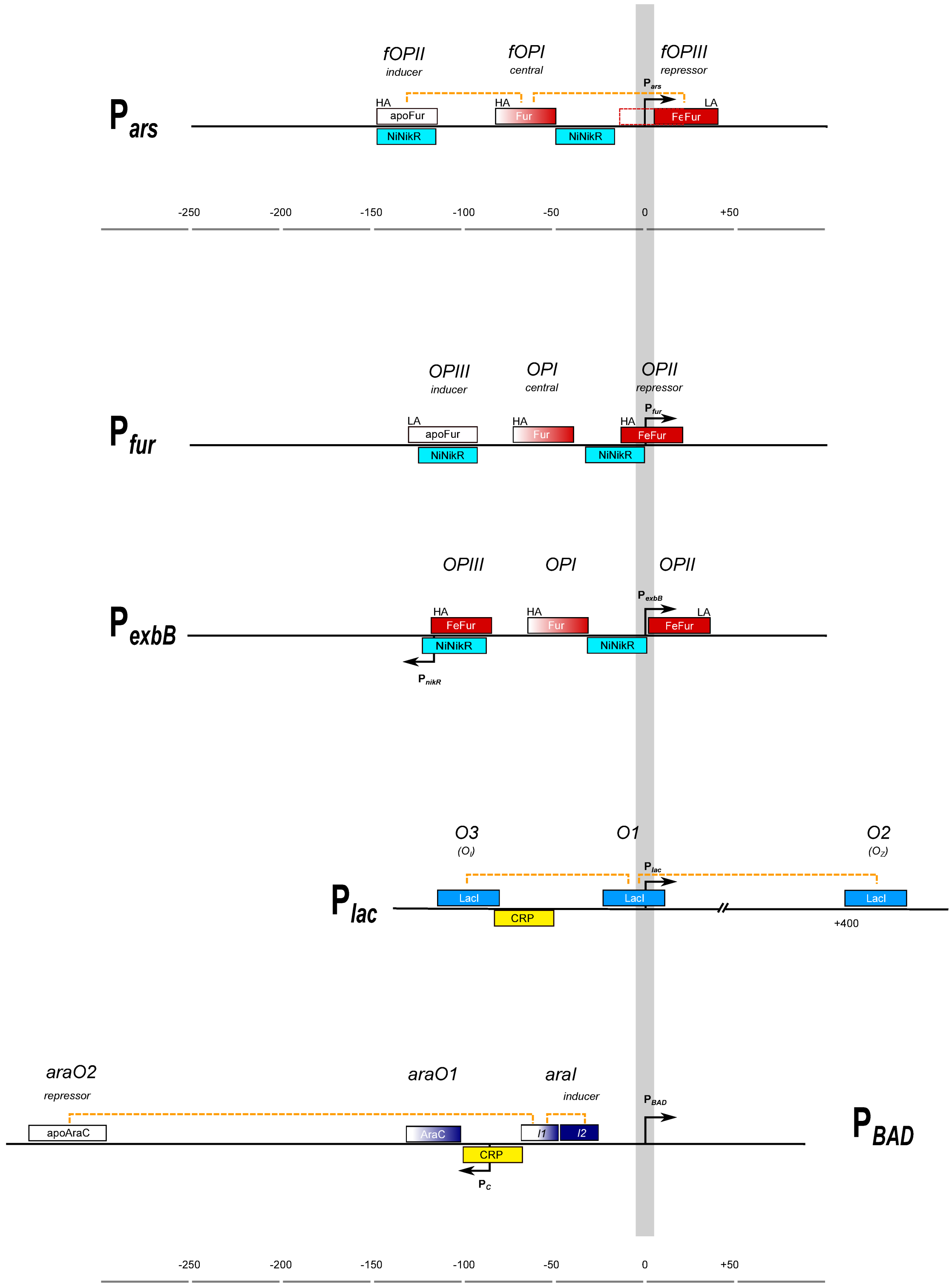

Supplementary Figure 5. Operator layout at complex *H. pylori* and *E. coli* promoters. Red boxes Fur operators; turquoise boxes: NikR operators; blue boxes: Lac or AraC operators; yellow boxes: CRP binding sites. Numbers indicate the position with respect to the TSS. Dashed lines indicate bona-fide short-range DNA looping or condensation events.

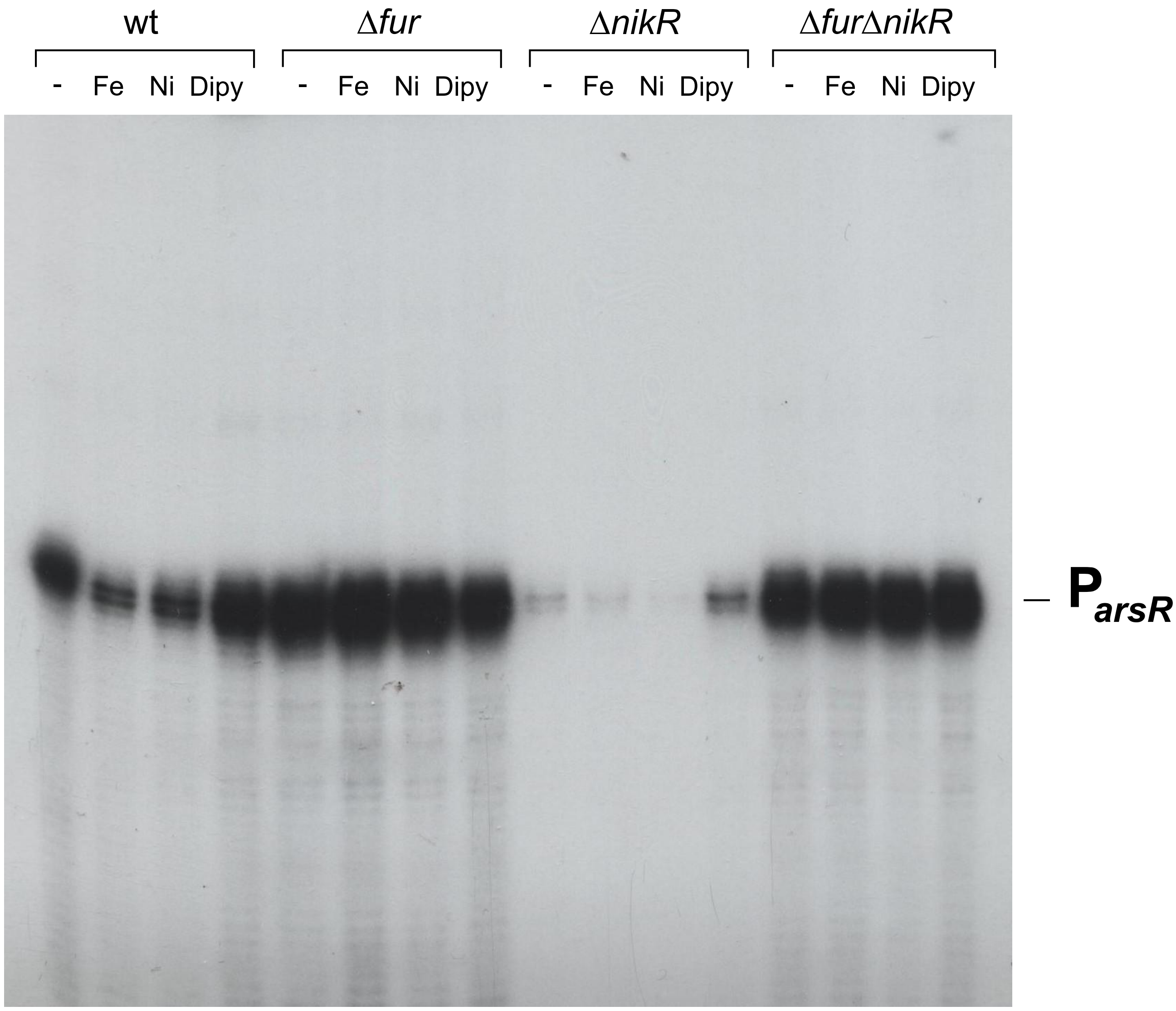

Supplementary Figure 6. Transcriptional responses of the P*arsR* promoter in response to metal ion treatment. Representative original gel of the primer extensions shown in Fig. 1. Total RNA was extracted from wild-type (wt), Δ*fur*, Δ*nikR* and Δ*fur*Δ*nikR* strains grown to exponential phase and treated for 15 minutes with 1 mM $(NH_4)_2Fe(SO_4)_2$ (Fe), 1 mM $NiSO_4$ (Ni) or 100 μM 2-2 dipyridyl (Dipy); untreated control RNA (-). Equal amounts of total RNA were reverse transcribed using oligo 166pe3.

SUPPLEMENTARY TABLES

Supplementary Table 1. Bacterial strains.

Name	Features	Reference
Helicobacter pylori G27	Clinical isolated, wild type	1
G27(*fur::km*)	G27 derivative in which the *fur* CDS (*HP1027*) from nt 25 to 434 was replaced by a Kanamicin resistence cassette, Km^R	2
G27(*nikR::km*)	G27 derivative in which the *nikR* CDS (*HP1338*) from nt 88 to 417 was replaced by a Kanamicin resistence cassette, Km^R	3
G27 *lux*	*vacA::aphA-3-luxCDABE*; G27 derivative carrying the *Campylobacter coli aphA-3* cassette and the promoterless *Photorhabdus luminescens luxCDABE* operon in the *vacA* locus, Km^R	4
G27 *lux* P*arsR*	G27 *lux* derivative, transformed with pVCC::P_{arsR} vector, Cp^R	This study
G27 *lux* P*arsR* nOPI*	G27 *lux* derivative, transformed with pVCC::P*arsR* nOPI* vector, Cp^R	This study
G27 *lux* P*arsR* ΔfOPII/nOPII	G27 *lux* derivative, transformed with pVCC::P*arsR* ΔfOPII/nOPII vector, Cp^R	This study
G27 *lux* P_{arsR} P*arsR* ΔfOPI	G27 *lux* derivative, transformed with pVCC::P*arsR* ΔfOPI, Cp^R	This study
G27 *lux* P_{arsR} nOPI* Δ*fur*	G27 *lux* P*arsR* nOPI* derivative in which the *fur* CDS (*HP1027*) from nt 25 to 434 was replaced by a Kanamicin resistence cassette, Cp^R, $Km^{R.}$	This study
G27 *lux* P*arsR* nOPI* ΔfOPIII	G27 *lux* derivative, transformed with pVCC::P*arsR* nOPI* ΔfOPIII, Cp^R, $Km^{R.}$	This study

Supplementary Table 2. Oligonucleotides

Name	5'-sequence-3'
166pe3	ATTAACTCCTTCAATGATTTCTG
166*Alida*	CGATTAGAATCTTTGGAGC
LuxRT FW	TTGGCAGATGTGTGTACCTTC
LuxRT RV	TGATGACTCCCAAGGAAAAATAG
Lux C3	GAGTCATTCAATATTGGCAG
ParsR_For	GCTTAATGCGCCGCTACAG
ParsR_Rev	TGCAGCTGGCACGACAGG
ΔOPIII FW	GTTTTTAAAGTTGCAGAAATCATTGAAGG
ΔOPIII RV	GGACTAATTCTAGCATTCCCTATTAAAGATACC
ParsRmut_For	CTTTAATTTTTAAGGATCCATGAAAACAAAG
ParsRmut_Rev	CTTTGTTTTCATGGATCCTTAAAAATTAAAG
ParsRins_For	CGCGGATCCACCGATGCCCTTGAGAGCC
ParsRins_Rev	GCGGGATCCTCATGCGCACCCGTGGGG

Supplementary Table 3. Plasmids

Name	Features	Reference
pVCC	Suicide transformation vector that allows transcriptional and translational fusions of the *lux* reporter in the *H. pylori* acceptor strain G27 *lux*, Ap^R, Cp^R	4
pVCC::P_{arsR}	pVCC derivative, encompassing the wild-type *arsR* promoter from nt -203 to + 60, Ap^R, Cp^R	This study
pVCC::P*arsR* nOPI* ΔfOPIII	P*arsR* nOPI* derivative, deleted of the low affinity fOPIII Fur operator, from positions -2 to +23 using the mutagenic primer couple ΔOPIII FW/RV, Ap^R, Cp^R	This study
pVCC::P*arsR* nOPI*	pVCC derivative, encompassing the mutant P*arsR* nOPI* promoter, Ap^R, Cp^R	This study

pVCC::P*arsR* ΔfOPII/nOPII	pVCC derivative, encompassing the mutant P*arsR* ΔfOPII/nOPII promoter, Ap^R, Cp^R	This study
pVCC::P*arsR* ΔfOPI	pVCC derivative, encompassing the mutant P*arsR* ΔfOPI promoter, Ap^R, Cp^R	This study
PGEM-T- Easy	Cloning vector, Ap^R	Promega
pET15b*Fur*	pET15b derivative, containing the CDS of the *fur*.gene, Ap^R.	2
pGEM-P*arsR*	pGEM-T easy derivative, contains the wild-type *arsR* promoter from -203 to +61, Ap^R	This study
pGEM-P*arsR2*	pGEM-P*arsR* derivative with a 315 bp spacer between fOPI and fOPII, Ap^R	This study

Supplementary Table 4. Synthetic promoter fragments used to engineer the mutant P*arsR* reporter constructs.

Name	Features
P*arsR* nOPI*	Fragment of the *arsR* promoter from -203 to + 60, containing the ATA →GGG substitution of from nt -18 to -20.
P*arsR* ΔfOPII/nOPII	Fragment of the *arsR* promoter from -203 to + 60, deleted of the distal apo-Fur and NikR overalpping operators, from position -114 to -144.
P*arsR* ΔfOPI	Fragment of the *arsR* promoter from -203 to + 60, deleted of the central Fur operator fOPI, from position -80 to -48.

SUPPLEMENTARY REFERENCES

1. Xiang, Z. *et al.* Analysis of expression of CagA and VacA virulence factors in 43 strains of Helicobacter pylori reveals that clinical isolates can be divided into two major types and that CagA is not necessary for expression of the vacuolating cytotoxin. *Infect. Immun.* **63,** 94–98 (1995).
2. Delany, I., Pacheco, A. B., Spohn, G., Rappuoli, R. & Scarlato, V. Iron-dependent transcription of the frpB gene of Helicobacter pylori is controlled by the Fur repressor protein. *J. Bacteriol.* **183,** 4932–4937 (2001).
3. Danielli, A. *et al.* Growth phase and metal-dependent transcriptional regulation of the fecA genes in Helicobacter pylori. *J. Bacteriol.* **191,** 3717–3725 (2009).
4. Vannini, A. *et al.* A convenient and robust in vivo reporter system to monitor gene expression in the human pathogen Helicobacter pylori. *Appl. Environ. Microbiol.* **78,** 6524–6533 (2012).
